# Recall of pre-existing cross-reactive B cell memory following Omicron BA.1 breakthrough infection

**DOI:** 10.1126/sciimmunol.abq3511

**Published:** 2022-05-12

**Authors:** Chengzi I. Kaku, Alan J. Bergeron, Clas Ahlm, Johan Normark, Mrunal Sakharkar, Mattias N. E. Forsell, Laura M. Walker

**Affiliations:** ^1^ Adimab, LLC, Lebanon, NH 03766, USA; ^2^ Norris Cotton Cancer Center, Dartmouth Hitchcock Medical Center, Lebanon, NH 03766, USA.; ^3^ Department of Microbiology and Immunology, Dartmouth College, Hanover, NH 03755, USA.; ^4^ Division of Immunology, Department of Clinical Microbiology, Umea University, Umea,; Sweden.; ^5^ Adagio Therapeutics, Inc., Waltham, MA 02451, USA.

## Abstract

Understanding immune responses following severe acute respiratory syndrome coronavirus 2 (SARS-CoV-2) breakthrough infection will facilitate the development of next-generation vaccines. Here, we profiled spike (S)-specific B cell responses following Omicron/BA.1 infection in mRNA-vaccinated donors. The acute antibody response was characterized by high levels of somatic hypermutation (SHM) and a bias toward recognition of ancestral SARS-CoV-2 strains, suggesting the early activation of vaccine-induced memory B cells (MBCs). BA.1 breakthrough infection induced a shift in B cell immunodominance hierarchy from the S2 subunit, which is highly conserved across SARS-CoV-2 variants of concern (VOCs), and toward the antigenically variable receptor binding domain (RBD). A large proportion of RBD-directed neutralizing antibodies isolated from BA.1 breakthrough infection donors displayed convergent sequence features and broadly recognized SARS-CoV-2 VOCs. Together, these findings provide insights into the role of pre-existing immunity in shaping the B cell response to heterologous SARS-CoV-2 variant exposure.

## Teaser

BA.1 breakthrough infection activates pre-existing memory B cells with broad activity against SARS-CoV-2 variants.

## INTRODUCTION

mRNA-based COVID-19 vaccines demonstrated a high degree of protective efficacy against the original SARS-CoV-2 Wuhan-1 strain in clinical studies (*1*, *2*). However, waning vaccine-induced immunity combined with the continued emergence of resistant SARS-CoV-2 variants has significantly undermined vaccine effectiveness (*3*–*5*). In particular, the Omicron variant (B.1.1.529/BA.1) and its sub-lineages (e.g., BA1.1 and BA.2) display a striking degree of antibody evasion, thus eroding vaccine efficacy against this variant of concern (VOC) and allowing it to rapidly displace Delta and drive a global surge in COVID-19 caseloads (*6*–*11*).

Understanding the role of antigenic imprinting in shaping the B cell response to antigenically drifted SARS-CoV-2 variants will be critical for the development of next-generation COVID-19 vaccines. Previous studies have shown that Delta or Omicron breakthrough infection boosts serum neutralizing activity against both the Wuhan-1 vaccine strain and the infecting variant, potentially suggesting recall of cross-reactive vaccine-induced MBCs (*12*–*14*). However, the specificities, functions, and genetic features of the antibodies mediating this response remain poorly defined. To address these questions, we investigated S-specific serological and peripheral B cell responses in a cohort of mRNA-vaccinated individuals who had recently experienced BA.1 breakthrough infections.

## RESULTS

### Patients and sample collection

**Fig. 1.**
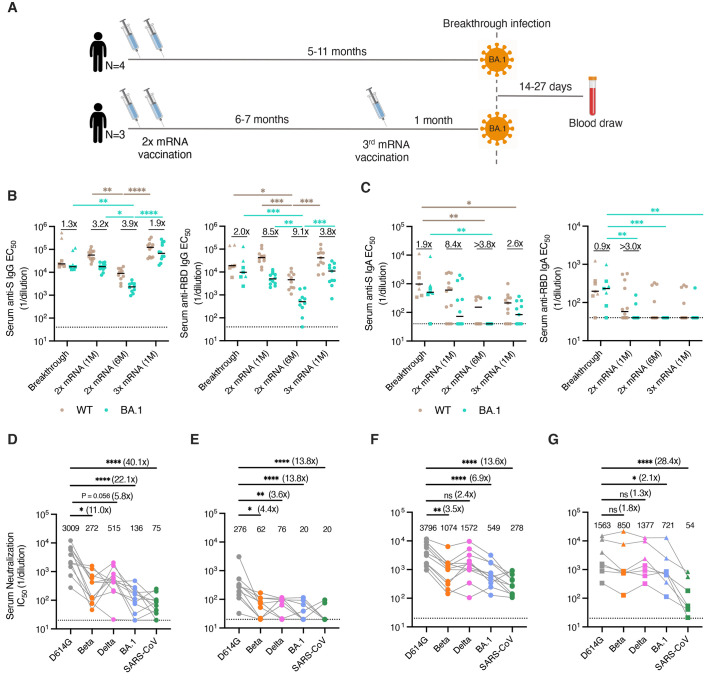
**Serum binding and neutralizing activity following BA.1 breakthrough infection. (A)** Vaccination, infection, and blood draw timelines. **(B-C)** Serum (B) IgG and (C) IgA reactivity with recombinant WT and BA.1 (left) Hexapro-stabilized S proteins and (right) RBDs following BA.1 breakthrough infection. Serum samples from uninfected/vaccinated donors at one month or six months following primary vaccination (2x mRNA) or one month following booster mRNA vaccination (3x mRNA) are shown for comparison. The fold change in median EC_50_ against BA.1 relative to D614G is shown above each paired set of measurements. Black bars represent median binding EC_50_ titers. Dotted lines represent the lower limit of detection. **(D-G)** Serum neutralizing activity against SARS-CoV-2 D614G, Beta, Delta, and BA.1 and SARS-CoV (D) one month after primary mRNA vaccination (n=12), (E) six months after primary mRNA vaccination (n=10), (F) one month after mRNA booster vaccination (n=11), and (G) 14 to 27 days after BA.1 breakthrough infection (n=7), as measured using an MLV-based pseudovirus neutralization assay. Plotted values represent serum neutralizing IC_50_ titers and values shown above the data points indicate the median IC_50_ titer. The fold change in IC_50_ titer for each virus relative to D614G is shown in parentheses. Breakthrough infection donors infected after primary mRNA vaccination (n=4) are shown as squares and those infected after mRNA booster vaccination (n=3) are shown as triangles. Statistical comparisons were determined by (B-C) two-sided Kruskal-Wallis test with Dunn's multiple comparisons or (D) Friedman's test with multiple comparisons. 1M, one month; 6M, six months; EC_50_, 50% effective concentration; IC_50_, 50% inhibitory concentration; WT, wild type. *P < 0.05, **P < 0.01, ***P < 0.001, ****P < 0.0001.

We recruited seven mRNA (mRNA-1273 or BNT162b2)-vaccinated individuals residing in the Northastern region of the United States who experienced SARS-CoV-2 breakthrough infections between December 30, 2021 and Jan 19, 2022 (Table S1). All donors tested positive for SARS-CoV-2 by RT-PCR and experienced asymptomatic or mild disease. Although we were unable to obtain viral samples for whole genome sequencing, SARS-CoV-2 variant surveillance data indicates that the BA.1 variant accounted for the vast majority of infections in the Northeastern United States during this time period (Fig. S1). Breakthrough infections occurred either 5-11 months after completing a primary mRNA vaccination series (n=4) or one month after an mRNA booster dose (n=3). To study the acute B cell response induced by breakthrough infection, we collected serum and peripheral blood mononuclear cell (PBMC) samples 14 to 27 days following PCR-confirmed infection (Fig. 1A).

### Serum antibody responses following BA.1 breakthrough infection

We evaluated serum IgG and IgA responses to recombinant prefusion-stabilized Wuhan-1/wild type (WT) and BA.1 S proteins and RBD subunits following breakthrough infection. For comparison, we also assessed serum antibody responses in a separate cohort of previously uninfected individuals who had completed their primary vaccination series either one- or six-months prior to sampling or a third mRNA booster dose one month prior to sampling (Table S2). BA.1 breakthrough infection donors exhibited similar (within two-fold) serum IgG binding titers to BA.1 and WT S and RBD (Fig. 1B). In contrast, uninfected/mRNA vaccinated donors displayed two- to four-fold and four- to nine-fold reduced serum IgG binding to full-length BA.1 S and BA.1 RBD, respectively, relative to WT (Fig. 1B). Furthermore, breakthrough infection donors exhibited higher serum IgA binding titers to both WT and BA.1 RBDs relative to uninfected/vaccinated donors, although this did not reach statistical significance for WT RBD due to the increased variability in IgA responses and small sample sizes (Fig. 1C). These results are consistent with previous studies documenting enhanced serum IgA responses following breakthrough infection with Delta (Fig. 1C) (*15*, *16*).

Next, we assessed the samples for serum neutralizing activity against an ancestral SARS-CoV-2 strain (D614G), as well as BA.1, Delta, and Beta VOCs using a murine leukemia virus (MLV)-based pseudovirus assay. Consistent with prior studies, serum samples obtained from uninfected/vaccinated donors showed 3.5- to 11-fold and 7- to 22-fold lower neutralizing titers against Beta and BA.1, respectively, relative to D614G (Fig. 1D-F and Fig. S2). In contrast, serum samples from BA.1 breakthrough infection donors displayed similar (within two-fold) neutralizing titers against D614G and all VOCs tested, suggesting that BA.1 breakthrough infection broadens the serum neutralizing antibody response (Fig. 1G). To determine whether this breadth of activity extended to other sarbecoviruses, we tested the serum samples for neutralizing activity against SARS-CoV, which revealed similarly low serum neutralizing titers in BA.1 breakthrough donors and uninfected/vaccinated individuals (Fig. 1D-G). Thus, BA.1 breakthrough infection appears to induce broad responses to SARS-CoV-2 VOCs but not more antigenically distant sarbecoviruses.

### Cross-reactivity of memory B cells induced by BA.1 breakthrough infection

**Fig. 2.**
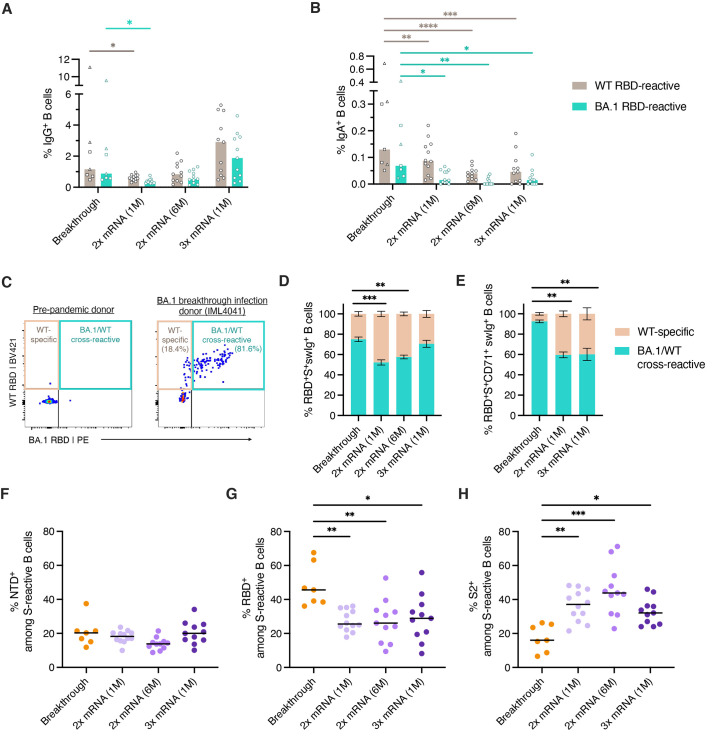
**SARS-CoV-2 S-specific B cell responses induced by BA.1 breakthrough infection. (A-B)** Frequency of circulating B cells that recognize recombinant WT and BA.1 RBDs among (A) IgG^+^ and (B) IgA^+^ B cells in BA.1 breakthrough infection donors (n=7) and uninfected/vaccinated donors at one month (n=12) or six months (n=11) following primary vaccination (2x mRNA) or one month following booster mRNA vaccination (3x mRNA, n=11), as measured by flow cytometry. Bars indicate median frequencies. Donors with breakthrough infections occurring after primary mRNA vaccination are shown as squares and those infected after booster mRNA vaccination are shown as triangles. **(C)** Representative fluorescence-activated cell sorting (FACS) gating strategy used to identify RBD-directed B cells that are WT-specific or WT/BA.1 cross-reactive, shown for a pre-pandemic donor and a breakthrough infection donor. Percentages of WT-specific and WT/BA.1 cross-reactive B cells out of total RBD-reactive cells are shown in parentheses. **(D-E)** Mean proportions of RBD-reactive B cells that bind WT and/or BA.1 RBDs among (D) total S^+^swIg^+^ B cells or (E) S^+^swIg^+^ CD7^+^ B cells. Error bars represent standard errors of the mean. A median of 65 RBD-specific B cells (ranging 12 to 310 cells) were collected from each donor for this analysis. Samples collected six months following mRNA vaccination were excluded from this analysis due to low numbers of RBD-specific CD71^+^ cells at this time point. **(F-H)** Percentage of S-reactive swIg^+^ B cells that target the (F) NTD, (G) RBD, and (H) Hexapro-stabilized S2 subunits. Black bars represent median percentages. For breakthrough infection donors, this analysis was restricted to S^+^swIg^+^CD71^+^ B cells to capture the activated response. 87 to 1721 S-reactive B cells were collected from each donor for this analysis. Statistical comparisons were determined by (A-B) two-way ANOVA with subsequent Dunnett's multiple comparisons test or (D-H) two-sided Kruskal-Wallis test with Dunn's multiple comparisons. 1M, one month; 6M, six months; swIg, class-switched (IgG^+^ or IgA^+^) immunoglobulin; WT, wild type. *P < 0.05, **P < 0.01, ***P < 0.001, ****P < 0.0001.

We next evaluated the magnitude and cross-reactivity of the peripheral RBD-specific B cell response following BA.1 breakthrough infection. Despite the higher serum neutralizing titers to BA.1 in breakthrough infection donors relative to uninfected/mRNA vaccinated individuals, the two cohorts showed similar frequencies of WT- and BA.1-RBD-reactive IgG^+^ B cells (Fig. 2A and Fig. S3A). The limited magnitude of the circulating IgG^+^ B cell response following BA.1 breakthrough infection may be due to localization of antigen in the upper respiratory tract during mild and asymptomatic infections. We also compared the frequencies of RBD-specific IgA^+^ B cells in breakthrough infection donors and uninfected/vaccinated individuals. In uninfected/vaccinated donors, WT and BA.1 RBD-reactive B cells represented medians of 0.04 to 0.087% and 0 to 0.015% of total IgA^+^ B cells, respectively (Fig. 2B). In contrast, breakthrough infection donors mounted significantly higher magnitude IgA responses to the RBD, with WT and BA.1 RBD-specific IgA^+^ B cells accounting for a median of 0.13% (ranging 0.05 to 0.7%) and 0.069% (ranging 0.025 to 0.4%), respectively, of the total IgA^+^ B cell population (Fig. 2B). The results were similar for breakthrough infections that occurred after both two- or three-dose mRNA vaccination (Fig. S4). We conclude that BA.1 breakthrough infection induces similar IgG^+^ B cell responses and higher magnitude IgA^+^ B cell responses to the BA.1 RBD relative to both two- and three-dose mRNA vaccination.

To investigate the impact of pre-existing vaccine-induced immunity on the B cell response to BA.1 breakthrough infection, we enumerated B cells that displayed WT/BA.1 RBD cross-reactivity in both BA.1 breakthrough donors and uninfected/mRNA vaccinated individuals (Fig. 2C and Fig. S3A). At one month post primary mRNA vaccination, only 48% of total RBD-directed B cells displayed cross-reactivity with BA.1 (Fig. 2D and Fig. S5A). The proportion of WT/BA.1 RBD cross-reactive B cells increased to 57% at 6-months post vaccination and to 70% following mRNA booster immunization, which is consistent with the evolution of anti-SARS-CoV-2 antibody breadth over time (Fig. 2D) (*17*–*19*). Following breakthrough infection, BA.1/WT RBD cross-reactive B cells constituted 65-83% of total anti-RBD B cells, with the remaining 17-35% only showing reactivity with the WT probe (Fig. 2D). Because WT RBD-specific B cells may represent resting MBCs induced by vaccination but not activated by BA.1 infection, we also performed this analysis on recently activated B cells expressing the activation/proliferation marker CD71 (Fig. S3B) (*20*). Consistent with previous studies demonstrating prolonged B cell activation following primary SARS-CoV-2 infection, 54 to 78% of RBD-specific B cells expressed CD71 (Fig. S5B) (*21*). The vast majority (87 to 98%) of these recently activated B cells displayed BA.1/WT RBD cross-reactivity, supporting the epidemiological data suggesting BA.1 as the breakthrough variant. In contrast, the proportion of cross-reactive B cells remained unchanged (averaging ~60%) in uninfected/mRNA vaccinated individuals after gating on CD71 expression (Fig. 2E and Fig. S5C). We were unable to detect BA.1-specific B cells that lacked WT cross-reactivity in any donors following BA.1 breakthrough infection, suggesting limited induction of de novo B cell responses at this early time point. We conclude that BA.1 breakthrough infection activates B cells that display cross-reactivity with both BA.1 and the original Wuhan-1 vaccine strain.

### BA.1 exposure re-directed the B cell response toward the RBD

Given the increased antigenic divergence of the BA.1 RBD and N-terminal domain (NTD) relative to the more conserved S2 subunit, we evaluated whether heterologous BA.1 S exposure modified the immunodominance hierarchy of B cells targeting each subdomain (NTD, RBD and S2 subunits) within the S trimer. To calculate the proportion of full-length S-reactive B cells targeting each subdomain, we stained B cells with differentially labeled tetramers of full-length S, RBD, NTD, and prefusion-stabilized S2 (Fig. S3C). In the uninfected/vaccinated cohort, class-switched B cells targeting the NTD, RBD, and S2 subdomains comprised 18%, 25%, and 37% of the total S-directed response, respectively, and these proportions remained largely unchanged at six months post primary vaccination and after mRNA booster immunization (Fig. 2F-H). In contrast, we observed significantly higher proportions of RBD-directed B cells among donors with breakthrough infection, ranging from 35-63% (median=46%) of the total activated (CD71^+^) S-specific B cell response (Fig. 2G). Furthermore, S2-reactive B cells comprised a smaller fraction (median=16%) of the S-specific response in breakthrough donors relative to uninfected/vaccinated individuals (medians 32 to 44%) (Fig. 2H). This modified pattern of immunodominance was observed in donors experiencing BA.1 breakthrough infection following both second and third dose mRNA vaccination (Fig. S6). In summary, BA.1 breakthrough infection appears to re-direct the B cell response from the S2 subunit to the RBD.

### Recall of highly mutated, cross-reactive memory B cells following BA.1 breakthrough infection

**Fig. 3.**
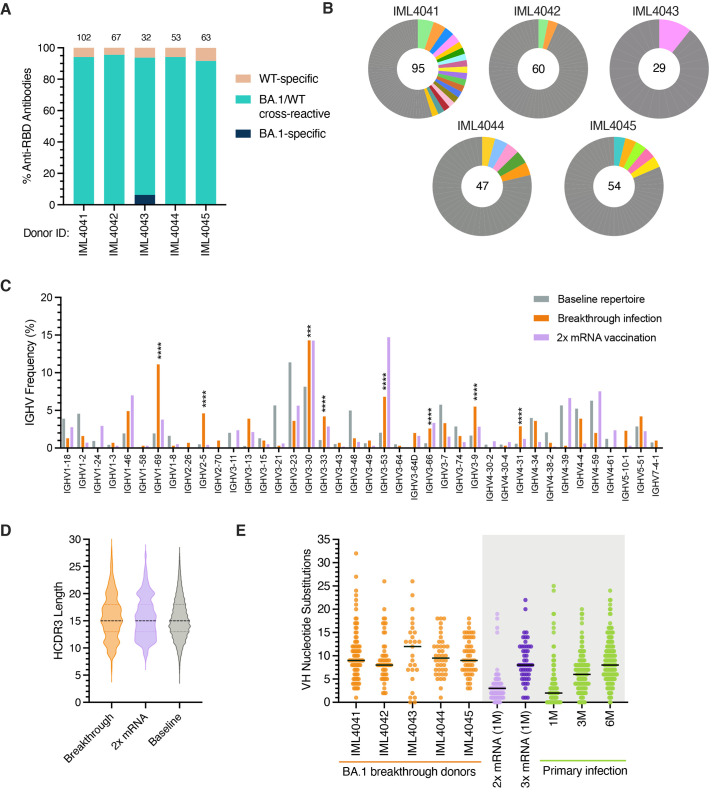
**Sequence features of RBD-directed monoclonal antibodies isolated from BA.1 breakthrough infection donors. (A)** Proportion of antibodies that bind recombinant WT and/or BA.1 RBD antigens from each donor, as determined by IgG binding via biolayer interferometry (BLI). Antibodies were isolated from breakthrough infection donors after two-dose vaccination (IML4041 through IML4044) or three-dose vaccination (IML4045). The number of antibodies isolated from each donor is indicated at the top of each bar. **(B)** Clonal lineage analysis of BA.1-reactive B cells. Clonally expanded lineages are represented as colored slices, with each differentially colored slice representing a separate lineage and the size of the slice proportional to the lineage size. Unique clones are combined and shown as a single grey segment. The total number of antibodies is shown in the center of each pie. **(C)** Germline IGHV gene usage frequencies among anti-RBD antibodies derived from breakthrough infection donors. Anti-RBD antibodies isolated from mRNA-vaccinated donors (purple bars) were obtained from the CoV-AbDab database (*45*). Unselected (baseline) memory B cell repertoires (grey bars) are included for reference (*22*). **(D)** Distribution of HCDR3 amino acid lengths in BA.1-reactive antibodies. Antibodies isolated from two-dose mRNA vaccinated individuals (from CoV-AbDab) and baseline human repertoire antibodies are shown for reference (*22*, *45*). The dotted black line represents the median, and the lower and upper lines represent the first and third quartile, respectively. **(E)** Somatic hypermutation levels are shown as the number of VH nucleotide substitutions in antibodies isolated from each breakthrough infection donor (orange), with medians shown by black bars. Antibodies isolated one month after two-dose mRNA vaccination (light green); one month after three-dose mRNA vaccination (dark green); and one, three, or six months after primary D614G infection (purple) are shown for comparison (*21*). Statistical significance was determined by Fisher's exact test compared to the baseline repertoire. 1M, one month; 3M, three months; 6M, six months; CDR, complementarity determining region; IGHV, immunoglobulin heavy variable domain; VH, variable heavy chain; WT, wild type. ***P < 0.001, ****P < 0.0001.

To characterize the molecular features of anti-RBD antibodies elicited by BA.1 breakthrough infection, we single-cell sorted 410 class-switched RBD^+^ B cells from five breakthrough infection donors (four donors infected after two-dose vaccination and one infected after three-dose vaccination) and expressed 317 natively paired antibodies as full-length IgGs (32 to 102 antibodies per donor) (Fig. S7). Despite sorting with a mixture of WT and BA.1 RBDs, over 90% of the IgGs displayed BA.1 RBD reactivity (Fig. 3A). In addition, index sorting analysis revealed that all antibodies derived from CD71^+^ B cells recognized BA.1, suggesting that the limited number of WT-specific antibodies likely originated from resting MBCs elicited by vaccination (Fig. S8A). We identified BA.1-specific antibodies that lacked WT cross-reactivity in only one donor, which represented 6% of their anti-RBD repertoire, further suggesting limited induction of de novo responses at this time point (Fig. 3A).

Sequence analysis revealed a relatively high degree of clonal diversity within the BA.1 RBD-reactive antibody repertoire, with 7-45% of antibodies belonging to expanded clonal lineages (Fig. 3B). We observed a significant over-representation of heavy chain germline genes IGHV1-69, 2-5, 3-30, 3-53, 3-66, and 3-9 in BA.1 breakthrough infection repertoires relative to the baseline human repertoire (Fig. 3C and Table S3) (*22*). Notably, IGHV3-53, 3-66, 3-30, and 3-9 germline genes are over-represented in the antibody response to ancestral SARS-CoV-2 strains, but IGHV1-69 and 2-5 appeared to be unique to the BA.1 breakthrough response (Fig. 3C) (*23*–*25*). The BA.1 RBD-reactive antibodies displayed a similar HCDR3 length distribution compared to the baseline repertoire (Fig. 3D). In support of an MBC origin, 95-100% of antibodies derived from each donor contained somatic mutations, and median levels of SHM ranged from 8 to 13 VH nucleotide substitutions per donor (Fig. 3E). While we were unable to collect paired pre-infection samples, we compared the levels of SHM in BA.1-reactive antibodies with those isolated from separate cohorts of individuals following vaccination or primary infection. BA.1-reactive antibodies displayed higher VH SHM loads than those observed in antibodies isolated one month following two-dose mRNA vaccination (median = 3 substitutions) and similar to that observed in antibodies isolated six months following primary infection (median = 8 substitutions) or one month following three-dose mRNA vaccination (median = 8 substitutions), consistent with prolonged antibody affinity maturation over time and the time elapsed from vaccination to breakthrough infection (Fig. 3E) (*21*, *26*, *27*). In contrast to the highly mutated WT/BA.1 cross-reactive antibodies, the two BA.1-specific antibodies had zero to one somatic mutation, suggesting recruitment from the naïve B cell population (Fig. S8B). Notably, MBCs activated by BA.1 infection displayed similar levels of SHM relative to resting WT-specific B cells isolated from the same individuals, suggesting a lack of further B cell affinity maturation following breakthrough infection, at least at this early time point (Fig. S8B). We conclude that the acute B cell response induced by BA.1 breakthrough infection is dominated by highly mutated clones that cross-react with both WT and BA.1 RBDs.

### BA.1 breakthrough infection expands pre-existing RBD-directed B cells with broad activity against SARS-CoV-2 VOCs

**Fig. 4.**
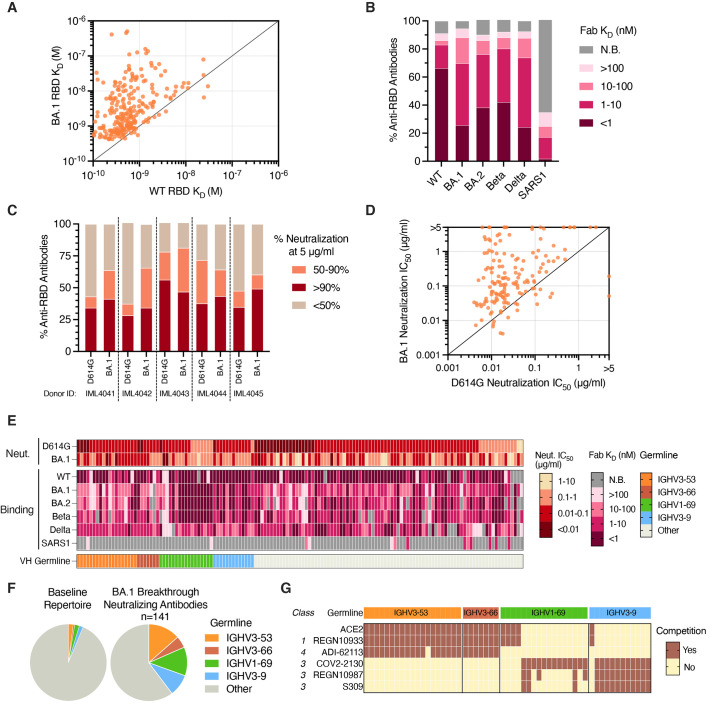
**Binding and neutralization properties of anti-RBD antibodies isolated following BA.1 breakthrough infection. (A)** Fab binding affinities for recombinant WT and BA.1 RBD antigens, as measured by BLI. Fabs with no detectable binding activity or with binding kinetics that could not be fit to a 1:1 binding model were excluded. **(B)** Proportion of BA.1-reactive antibodies with the indicated binding affinities for SARS-CoV-2 VOC RBDs and SARS-CoV RBD, as measured by BLI. Antibodies with weak binding affinities that could not be fit to a 1:1 binding model are shown as >100 nM, and antibodies with no detectable Fab binding, including those that bind only avidly, are indicated as non-binding (N.B.). **(C)** Proportion of antibodies from each donor with the indicated levels of neutralizing activity against MLV-SARS-CoV-2 D614G and BA.1 at a concentration of 5 μg/ml. **(D)** MLV-SARS-CoV-2 D614G and BA.1 neutralization IC_50_s for antibodies that displayed >90% neutralization against D614G and/or BA.1 at a concentration of 5 μg/ml. **(E)** Heatmap showing neutralization potency and binding breadth of BA.1 neutralizing antibodies. The bottom bar shows convergent IGHV germline gene families. Antibodies with weak binding affinities that could not be fit to a 1:1 binding model are shown as >100 nM, and antibodies with no detectable Fab binding are indicated as non-binding (N.B.). **(F)** Pie charts showing convergent germline gene usage among (right) BA.1-neutralizing antibodies compared to the (left) baseline human antibody repertoires (*22*). **(G)** Competitive binding profiles of BA.1 neutralizing antibodies utilizing convergent IGHV germline genes, as determined by BLI sandwich competition assay using ACE2 and the indicated comparator antibodies. Fab, antigen binding fragment; IC_50_, 50% inhibitory concentration; IGHV, immunoglobulin gene heavy variable; K_D_, equilibrium dissociation constant; N.B., non-binding; WT, wild type.

To further evaluate the binding properties of the BA.1 RBD-reactive antibodies, we measured their monovalent binding affinities for SARS-CoV-2 WT, BA.1, BA.2, Beta, and Delta RBDs and the SARS-CoV RBD. Seventy percent (204/293) of RBD-directed antibodies bound with high affinity (K_D_<10 nM) to both BA.1 and WT RBDs, supporting selection from an affinity matured B cell population (Fig. 4A). However, the majority (70%) of the antibodies displayed higher affinity binding (>2-fold) to WT (median K_D_ = 0.6 nM) relative to BA.1 (median K_D_ = 2.3 nM), providing further evidence that BA.1 breakthrough infection re-activates pre-existing vaccine-induced MBCs (Fig. 4A). In contrast to vaccine-induced anti-RBD antibodies, which often show reduced activity against the Beta VOC, only a minority (<5%) of antibodies derived from BA.1 breakthrough donors displayed loss of binding to Beta relative to WT (Fig. 4B and Fig. S8C) (*11*, *19*). This difference in antibody binding cross-reactivity is likely due to the presence of shared mutations within the Beta and BA.1 RBDs (E484K/A, K417N, and N501Y) (*18*). Overall, 82% (241/293) of anti-RBD antibodies isolated from breakthrough infection donors displayed monovalent binding to WT, Beta, Delta, BA.1, and BA.2 RBDs, suggesting that the majority of B cells activated by BA.1 breakthrough infection target conserved epitopes (Fig. 4B). In addition to epitope specificity, the breadth of the BA.1-activated response may also have been driven by high starting affinities for WT. Indeed, comparison with WT-specific antibodies showed that BA.1-reactive antibodies bound WT RBD with 5-fold higher median affinity despite similar levels of SHM, suggesting that both epitope specificity and starting affinity likely contributed to antibody breadth (Fig. S8D). Consistent with the weak serum neutralizing activity observed against SARS-CoV, less than 10% of RBD-targeting antibodies exhibited detectable monovalent binding to the SARS-CoV RBD (Fig. 4B). Thus, BA.1 breakthrough infection appears to preferentially expand high affinity B cells that broadly recognize SARS-CoV-2 variants but not more antigenically divergent sarbecoviruses.

Next, we screened the BA.1 RBD-reactive antibodies for neutralizing activity against D614G and BA.1. Twenty-eight to 56% and 34 to 49% of antibodies from each donor displayed >90% neutralizing activity against D614G and BA.1, respectively, at a concentration of 5 μg/ml (Fig. 4C). Titration of the neutralizing antibodies against D614G and BA.1 revealed that 45% (64/141) potently neutralized both viruses with IC_50_s less than 0.1 μg/ml (Fig. 4D). Consistent with their overall increased binding affinities for the WT RBD relative to BA.1, most of the neutralizing antibodies (78%) displayed higher potency against D614G (median IC_50_ = 0.020 μg/mL) compared to BA.1 (median IC_50_ = 0.13 μg/mL) (Fig. 4D). Notably, a large proportion of BA.1 neutralizing antibodies also displayed cross-reactivity with Delta (79%), Beta (90%), and BA.2 (86%) RBDs with affinities within 10-fold of BA.1 (Fig. 4E). A limited number of these VOC cross-reactive antibodies (5 out of 141) also neutralized SARS-CoV, with IC_50_s ranging from 0.039-0.35 μg/ml (Fig. S9). We thus conclude that BA.1 breakthrough infection elicits RBD-directed antibodies with broad activity against SARS-CoV-2 VOCs.

### BA.1-neutralizing antibodies display convergent sequence and binding properties

There are several “public” classes of neutralizing antibodies (Class 1-4) induced by SARS-CoV-2 infection and vaccination (*25*, *28*). To determine whether BA.1 breakthrough infection also elicited recurrent neutralizing antibody responses, we analyzed the sequence and binding features of the BA.1 neutralizing antibodies. Over 40% of all BA.1 neutralizing antibodies utilized one of four VH germline genes (IGHV3-53/66, IGHV1-69, and IGHV3-9) (Fig. 4F and Fig. S10). Similar to previously described IGHV3-53/66 antibodies isolated from infected and mRNA-vaccinated individuals, the BA.1 neutralizing IGHV3-53/66 antibodies possessed short HCDR3s (11-12 residues) and displayed competitive binding with ACE2, the class 1 mAb REGN10933, and the COVA1-16-like class 4 mAb ADI-62113 (*29*) (Fig. 4G, Fig. S11A, and Fig. S12). However, unlike vaccine-induced IGHV3-53/66 antibodies, which generally lack activity against SARS-CoV-2 variants containing substitutions at position K417 (e.g., Beta, Gamma, and BA.1), breakthrough infection-derived IGHV3-53/66 antibodies displayed broad reactivity with all VOCs tested and potently neutralized both D614G and BA.1 pseudoviruses (median IC_50_ = 0.016 and 0.051 μg/ml, respectively) (Fig. 4E and Fig. S11B) (*21*, *30*). Thus, BA.1-induced IGHV3-53/66-utilizing antibodies appear to recognize an antigenic site that is overlapping but distinct from previously described IGHV3-53/66 antibodies induced by infection and vaccination with ancestral SARS-CoV-2 strains.

Neutralizing antibodies utilizing the IGHV1-69 and IGHV3-9 germline genes also broadly recognized SARS-CoV-2 variants, including BA.2 (Fig. 4E). In contrast to IGHV3-53/66, these germline genes have not been shown to be over-represented in the human antibody response to primary SARS-CoV-2 infection or vaccination (*24*, *25*, *31*). Antibodies utilizing the IGHV1-69 germline gene segregated into two groups, one comprised of antibodies that targeted an ACE2- and REGN10933-competitive region and the other containing antibodies that recognized a non-ACE2 competitive site overlapping the COV2-2130 (class 3) epitope (Fig. 4G and Fig. S12B). Notably, >80% of the non-ACE2 competitive clones utilized the light chain IGLV1-40 gene and displayed highly similar LCDR3 sequences, suggesting a convergent mode of recognition (Fig. S11C and Fig. S11D). Finally, 12/13 IGHV3-9 antibodies recognized an epitope outside of the ACE2 binding site and competed with all three class 3 antibodies tested (S309, REGN10987, and COV2-2130), suggesting a binding mode distinct from the IGHV1-69 antibodies (Fig. 4G). Taken together, BA.1 breakthrough infection elicits multiple recurrent classes of anti-RBD antibodies with broad SARS-CoV-2 VOC recognition.

## DISCUSSION

A deep understanding of how pre-existing SARS-CoV-2 immunity shapes the B cell response to heterologous variant exposure is important for the development of variant-based booster vaccines. Here, we demonstrated that the acute B cell response to BA.1 breakthrough infection was primarily mediated by re-activated vaccine-induced memory B cell clones with broader SARS-CoV-2 VOC cross-reactivity than those elicited by infection or vaccination with ancestral SARS-CoV-2 strains. BA.1-reactive antibodies displayed high SHM loads and biased reactivity with the ancestral vaccination strain, providing strong evidence for the recall of memory B cells established by prior vaccination. In contrast, we observed limited induction of de novo BA.1-specific antibody responses at this timepoint, which is consistent with previous studies demonstrating the early expansion of cross-reactive antibodies following heterologous influenza vaccination (*32*). Longitudinal follow-up studies will be required to determine whether de novo BA.1-specific B cell responses appear at later timepoints, as observed in the context of influenza (*32*). Nevertheless, the early induction of cross-reactive B cell responses following BA.1 breakthrough infection suggests that heterologous variant exposure likely confers broad protection against SARS-CoV-2 VOCs, as supported by the robust BA.2 cross-protection observed among BA.1 breakthrough infected individuals (*33*, *34*).

To date, a limited number of studies have been published describing human antibody responses induced by heterologous SARS-CoV-2 variant exposure. Booster immunization of mRNA-1273-primed individuals with a Beta spike vaccine (mRNA-1273.351) did not induce broader neutralizing antibody responses relative to homologous WT boost (*35*). Similarly, heterologous Delta infection in mRNA-vaccinated individuals boosted neutralizing titers to Delta but not BA.1 (*14*). In contrast, COVID-19 vaccination induced broadly neutralizing antibody responses to SARS-CoV-2 VOCs and highly divergent sarbecoviruses in SARS-CoV convalescent individuals, which is likely due to recall of pre-existing cross-reactive B cells induced by an antigenically divergent SARS virus (*36*). Similarly, avian influenza H5N1 and H7N9 immunization has been shown to elicit broadly reactive anti-HA responses in individuals previously exposed to seasonal H1 and H3 influenza viruses (*37*, *38*). Thus, booster immunization with an antigenically divergent S protein, such as the hyper-mutated Omicron spike, may be a promising strategy for the elicitation of broadly neutralizing responses against future emerging VOCs.

Despite the relative conservation of the BA.1 S2 subunit compared with the RBD, BA.1 breakthrough infection preferentially boosted cross-reactive antibodies targeting the RBD. The molecular explanation(s) for the dampened antibody response to the S2 subunit remain to be determined but may be driven by increased serum antibody masking of the conserved S2 subunit relative to the more divergent RBD, resulting in limited S2 epitope accessibility for B cell targeting. Conversely, the extensive immune evasion of the BA.1 RBD likely resulted in substantially lower levels of serum antibody feedback, potentially enabling the activation of rare cross-reactive RBD-directed memory B cells. In support of this hypothesis, studies of malaria and influenza vaccination have demonstrated that serum antibody masking can modulate immunodominance patterns via the selective suppression of B cell responses to dominant epitopes and enhanced expansion of subdominant responses (*39*, *40*). In contrast, the B cell immunodominance hierarchy established by primary WT SAR-CoV-2 infection and vaccination remains stable following mRNA booster vaccination, suggesting that the immunodominance shift observed following BA.1 breakthrough infection is likely driven by heterologous exposure to an antigenically distinct variant rather than the route or number of exposures (*17*, *41*).

Finally, while the large majority of BA.1 breakthrough infection-induced antibodies did not neutralize more antigenically distal sarbecoviruses, we identified several monoclonal antibodies from BA.1 breakthrough infection donors that display broad activity against all SARS-CoV-2 VOCs described to date as well as SARS-CoV. Notably, many previously described neutralizing antibodies, including those targeting RBD epitopes conserved across sarbecoviruses, show reduced activity against one or more Omicron lineages (*42*). Thus, these rare broadly neutralizing antibodies represent promising candidates for therapeutic development and provide a framework for the development of vaccines that induce broadly neutralizing antibody responses.

## MATERIALS AND METHODS

### Study design.

Seven participants with BA.1 breakthrough infection were recruited with informed consent to participate in this study. SARS-CoV-2 infection was determined by positive results via both RT-PCR from a saliva sample and rapid antigen test from a nasal swab sample. All participants were previously immunized with two- or three-doses of an mRNA vaccine (BNT162b2 or mRNA-1273) and had no documented history of SARS-CoV-2 infection prior to vaccination. Venous blood samples were collected 14 to 27 days after their first SARS-CoV-2 positive test and separated to obtain plasma and peripheral blood mononuclear cells (PBMCs). Plasma samples were used to measure binding and neutralizing activity. PBMCs were used for the flow cytometric profiling the SARS-CoV-2 S-directed B cell response and for the isolation and characterization of RBD-directed monoclonal antibodies. Serum and B cell responses following breakthrough infection were compared with those of blood samples collected from a separate cohort of uninfected individuals at one month and six months after two-dose mRNA vaccination, as well as a third independent cohort of uninfected individuals one month after three-dose mRNA vaccination. Clinical and demographic characteristics are shown in Table S1 for breakthrough infection donors and Table S2 for uninfected/vaccinated donors. This study was unblinded and not randomized.

### Ethics permits and sample collection.

Breakthrough infection donors (n=7) and uninfected, two-dose vaccinated donors (n=12) participated with informed consent under the healthy donor protocol D10083, Immune Monitoring Core (DartLab) Laboratory at Dartmouth-Hitchcock Hospital (D-HH). Longitudinal samples were collected from uninfected/vaccinated individuals at one month (n=12) and six months (n=11) following the second mRNA dose. Uninfected, three-dose vaccinated participants (n=11) are enrolled in the clinical trial, CoVacc - Immune response to vaccination against Covid-19, an open multicenter phase IV study, was approved by the Swedish Ethics Review Authority (Dnr 2021-00055) and the Medical Products Agency Sweden. The study was registered at European Clinical Trials Database (EUDRACT Number 2021-000683-30) before the first patient was enrolled. Umeå University, Sweden served as trial sponsor and the Clinical Research Center, University Hospital of Northern Sweden was monitoring the study for regulatory compliance. Individuals were included after informed consent and data were stored in accordance with the EU General Data Protection Regulation. Detailed methodology for the isolation of plasma and PBMCs are described in the supplementary material.

### Recombinant SARS-CoV-2 S production.

To produce prefusion-stabilized WT SARS-CoV-2 HexaPro S, DNA encoding residues 1-1208 of the SARS-CoV-2 spike (GenBank NC NC_045512.2) with substitutions F817P, A892P, A899P, A942P, K986P, V987P, “GSAS” mutations from positions 682-685 and a C-terminal T4 fibritin motif, 8X HisTag and TwinStrepTag (SARS-CoV-2 S-2P) was cloned into a pcDNA3.4 vector. The following mutations were additionally cloned into the Omicron/BA.1 HexaPro S plasmid: A67V, Δ69-70, T95I, G142D, Δ143-145, Δ211, L212I, ins214EPE, G339D, S371L, S373P, S375F, K417N, N440K, G446S, S477N, T478K, E484A, Q493K, G496S, Q498R, N501Y, Y505H, T547K, D614G, H655Y, N679K, P681H, N764K, D796Y, N856K, Q954H, N969K, L981F. Plasmids were transiently transfected into FreeStyle HEK 293F cells (Thermo Fisher) using polyethylenimine following the manufacturer's directions. After one week of culture, the supernatants were harvested, and centrifuged to remove cellular debris. S protein preps were purified by Ni affinity chromatography and followed by size exclusion chromatography using the Superose 6 column (GE Healthcare) before concentrating and freezing at -80°C.

### Serum ELISAs.

Briefly, serum ELISAs were performed by coating 96-well half-area plates with recombinant SARS-CoV-2 antigens. Following overnight incubation, plates were blocked using 3% bovine serum albumin and then incubated in serial dilutions of human sera. Antigen-specific IgG and IgA were detected using anti-human IgG horseradish peroxidase (HRP) or anti-human IgA HRP, and binding signals were developed using TMB substrate solution. Detailed methodology can be found in the supplementary material.

### SARS-CoV-2 pseudovirus generation.

Single-cycle infection pseudoviruses were generated as previously described (*43*). Briefly, HEK293T cells seeded overnight in 6-well tissue culture plates were co-transfected with plasmids encoding SARS-CoV-2 spike, MLV luciferase reporter gene, and MLV gag/pol using Lipofectamine 2000 following the manufactuerer's recommendations. Culture supernatants containing SARS-CoV-2 S-pseudotyped MLV particles were harvested 48 hours post-transfection. Additional details are included in the supplementary material.

### Pseudovirus neutralization assay.

Briefly, SARS-CoV-2 S pseudotyped MLV viral stock was incubated with serial dilutions of monoclonal antibodies or heat-inactivated sera for 1 hour at 37°C with 5% carbon dioxide. Virus-antibody mixtures were subsequently added to a confluent monolayer of HeLa-hACE2 reporter cells in 96-well tissue culture plates and incubated for 48 hours. Viral infectivity was measured by cell lysis and detection of luciferase activity using the Luciferase Assay System (Promega). Experimental details are inlucded in the supplementary material.

### FACS analysis of SARS-CoV-2 S-specific B cell responses.

Antigen-specific B cells were detected using recombinant biotinylated antigens tetramerized with fluorophore-conjugated streptavidin (SA). Briefly, for detection of peripheral B cells that recognize WT and/or BA.1 RBD, B cells were stained with a mixture of recombinant WT and BA.1 HexaPro S and RBD tetramers. For determination of subdomain reactivities within the total S-specific B cell population, B cells were stained with antigen tetramers of WT and BA.1 HexaPro S, WT and BA.1 RBDs, WT and BA.1 NTDs, and WT Hexapro-stabilized S2. PBMCs were also labeled with anti-human antibodies targeting CD19, CD3, CD8, CD14, CD16, IgG, IgA, CD27, and CD71. Excess tetramers and antibody reagents were removed by washing, and PBMC samples were analyzed using a BD FACS Aria II (BD Biosciences). Experimental details are included in the supplementary material.

### Single B cell sorting.

Briefly, PBMCs were incubated with tetramerized recombinant WT and BA.1 RBD antigens (25 nM each) and a mixture of antibodies targeting human CD19, CD20, CD3, CD8, CD14, CD16, IgM, IgG, IgA, CD27, and CD71. Cells were washed two times to remove excess reagents and analyzed using a BD FACS Aria II (BD Biosciences). Class-switched B cells, defined as CD19^+^CD3^−^CD8^−^CD14^−^CD16^−^PI^−^IgM^−^ and IgG^+^ or IgA^+^, that specifically bound to the WT/BA.1 RBD mixture were single-cell index sorted into 96-well polystyrene microplates and frozen at -80°C prior to downstream processing. Additional details can be found in the supplementary material.

### Amplification and analysis of antibody variable genes.

Antibody variable gene mRNA transcripts (VH, Vk, Vλ) were amplified by RT-PCR as described previously (*21*). Briefly, cDNA was synthesized using SuperScript IV enzyme (ThermoFisher Scientific), followed by two rounds of nested PCRs. The second cycle of nested PCR added 40 base pairs of 5′ and 3′ homology to restriction enzyme-digested *S. cerevisiae* expression vectors to enable homologous recombination during transformation. PCR-amplified variable gene DNA was chemically transformed into competent yeast cells via the lithium acetate method and yeast were plated on selective amino acid drop-out agar plates (*44*). Transformed yeast colonies were picked for sequencing, recombinant antibody expression, and characterization.

For clonal lineage analysis, clonally expanded antibodies were defined by the following criteria: identical heavy and light chain germline genes, identical HCDR3 lengths, and >80% identical HCDR3 protein sequence.

### Expression and purification of IgG and Fab molecules.

Antibodies were expressed as human IgG1 via *S. cerevisiae* cultures, as described previously (*21*). Briefly, yeast cells were grown for IgG expression over 6 days, and the IgG-containing supernatant was subsequently harvested by centrifugation. Antibodies were purified by protein A-affinity chromatography, eluted with a solution of 200 mM acetic acid/50 mM NaCl (pH 3.5). The pH was then neutralized using 1/8^th^ volume of 2 M Hepes (pH 8.0)

Fab fragments were generated by incubating IgG with papain for 2 hours at 30°C. The reaction was terminated using iodoacetamide, and the mixture containing digested Fab and Fc was purified by Protein A agarose to remove Fc fragments and undigested IgG. Fabs present in the flow-through were further purified using CaptureSelect IgG-CH1 affinity resin (ThermoFisher Scientific) and eluted from the column using 200 mM acetic acid/50 mM NaCl (pH 3.5). Fab solutions were pH-neutralized using 1/8th volume 2 M Hepes (pH 8.0).

### Binding affinity measurements by biolayer interferometry.

Binding affinities were measured by biolayer interferometry (BLI) using a FortéBio Octet HTX instrument (Sartorius). Briefly, recombinant biotinylated antigens were loaded onto streptavidin biosensors and subsequently exposed to Fab or IgG fragments to measure the association rate. Sensors were next dipped into PBSF to measure the dissociation rate. Additional experimental details can be found in the supplementary material.

### Epitope binning by biolayer interferometry.

Antibody competition with recombinant human ACE2 and comparator antibodies for binding to SARS-CoV-2 RBD was determined by BLI. Briefly, for ACE2 competition experiments, test antibodies were captured onto anti-human IgG capture biosensors, followed by loading of recombinant RBD. Finally, biosensors were exposed to human ACE2 in solution to assess competitive binding. Antibody competition experiments were performed using the same method but with a different orientation: biosensors were coated with comparator antibodies (REGN10933, ADI-62113, COV2-2130, REGN10987, and S309) and then exposed to antibodies of interest in solution. Detailed experimental methods are included in the supplementary material.

### Statistics.

All statistical analyses were performed using GraphPad Prism (version 9.3.1). Detailed statistical results and exact p-values are shown in Table S4.

## Supplementary Materials

### Other Supplementary Material for this manuscript includes the following:

MDAR Reproducibility Checklist
