# Supplementary material for: Recall of pre-existing cross-reactive B cell memory following Omicron BA.1 breakthrough infection
Source: Sci Immunol. 2022 May 12:eabq3511. doi: 10.1126/sciimmunol.abq3511 (PMC9097882; doi:10.1126/sciimmunol.abq3511)
Supplement: Supplementary file 1 — Figs. S1 to S12 [file sciimmunol.abq3511_sm.pdf]

Science Immunology
AAAS

Supplementary Materials for

Recall of pre-existing cross-reactive B cell memory following Omicron BA.1 breakthrough infection

Chengzi I. Kaku *et al.*

Corresponding author: Laura M. Walker, lwalker@adagiotx.com

DOI: 10.1126/sciimmunol.abq3511

The PDF file includes:

Materials and Methods
Figs. S1 to S12
Tables S1 and S2

Other Supplementary Material for this manuscript includes the following:

MDAR Reproducibility Checklist
Tables S3 and S4

Supplementary Materials for

Recall of pre-existing cross-reactive B cell memory following Omicron BA.1 breakthrough infection

Chengzi I. Kaku, Alan J. Bergeron, Clas Ahlm, Johan Normark, Mrunal Sakharkar, Mattias N. E. Forsell, Laura M. Walker*

*Corresponding author. Email: lwalker@adagiotx.com

This PDF file includes:

Supplementary materials and methods.
Fig. S1. Prevalence of circulating SARS-CoV-2 variants.
Fig. S2. Serum neutralizing activity against SARS-CoV-2 VOCs and SARS-CoV.
Fig. S3. FACS gating strategy for SARS-CoV-2 antigen-specific B cell staining.
Fig. S4. Frequency of RBD-directed IgG and IgA B cells.
Fig. S5. Cross-reactivity of the BA.1 activated B cell response.
Fig. S6. B cell subdomain specificities following BA.1 breakthrough infection.
Fig. S7. FACS gating strategy for RBD-specific B cell sorting.
Fig. S8. Sequence and binding properties of BA.1 breakthrough infection-derived antibodies.
Fig. S9. Breadth of activity of SARS-CoV cross-neutralizing antibodies.
Fig. S10. IGHV germline gene usage among BA.1-neutralizing antibodies.
Fig. S11. Sequence and functional properties of convergent germline antibodies.
Fig. S12. Competitive binding assay schematic.
Table S1. BA.1 breakthrough infection donor characteristics.
Table S2. Uninfected/mRNA-vaccinated cohort characteristics.

Other Supplementary Material for this manuscript includes the following:

Table S3. Sequence, binding, and neutralization properties of isolated antibodies. (Excel datasheet)
Table S4. Raw data. (Excel datasheet)

Supplementary materials and methods.

Ethics permits and sample collection.

Blood samples among breakthrough infection donors and uninfected/two-dose vaccinated donors were collected at Dartmouth-Hitchcock Hospital. Venous blood was collected using BD Vacutainer® tubes with acid citrate dextrose (ACD), and plasma and PBMCs were isolated using a Ficoll 1077 (Sigma) gradient, washed, and counted with an anti-human CD45 stain on a volumetric flow cytometer. PBMC were frozen in 12.5% human serum and 10% DMSO diluted in RPMI-1040 and stored in liquid nitrogen until use. Plasma was isolated and frozen at -80 ˚C.

Venous blood was collected from uninfected/three-dose vaccinated donors at Umeå University. PBMCs and plasma isolated in BD EDTA Vacutainer® CPT™ tubes. PBMCs were frozen in 90% fetal calf serum supplemented with 10% DMSO and stored in liquid nitrogen until use. Plasma and serum were stored at -80 ˚C.

Serum ELISAs.

96-well half-area plates (Corning) were coated with the following recombinant antigens at a concentration of 5 µg/ml diluted in PBS: SARS-CoV-2 WT Hexapro-stabilized S, BA.1 Hexapro-stabilized S, WT RBD (Sino Biological, Cat #40592-V08B), BA.1 RBD (Acro Biosystems, Cat #SPD-C522e), WT NTD (Acro Biosystems, Cat #S1D-52H6), BA.1 NTD (Acro Biosystems, Cat #SPD-C522d), and Hexapro-stabilized S2 (Acro Biosystems, Cat #S2N-C52H5) antigens. Following overnight incubation at 4 ˚C, wells were washed with wash buffer (1X PBS with 0.05% Tween-20) and blocked with 75 µl 3% bovine serum albumin (BSA) in 1X PBS for 1 h at 37 °C. Coated wells were subsequently incubated with serial dilutions of human sera ranging from 1:40 to 1:1,310,720 in a solution of 0.1% BSA, 0.01% Tween-20 in 1X PBS for 1 h at 37 °C and then washed three times with wash buffer. To detect antigen-specific IgG and IgA, wells were incubated with either a 1:5000 dilution of anti-human IgG horseradish peroxidase (HRP; Jackson Immunoresearch Laboratories, Cat #109-036-098) or a 1:10,000 dilution of anti-human IgA HRP (Jackson Immunoresearch Laboratories, Cat #109-036-011) in 0.1% BSA, 0.01% Tween-20, 1X PBS for 1 h at 37 ˚C. Plates were then washed three times and developed with 25 µl of room temperature-equilibrated 1-Step™ Ultra TMB Substrate Solution (Thermo Fisher Scientific) for 5 min. The developing reaction was terminated by addition of 25 µl 4 N sulfuric acid. Absorbance was measured at 450 nm using a Spectramax microplate reader (Molecular Devices). Titration curves were fitted via non-linear regression to determine the 50% effective concentration (EC_{50}) in GraphPad Prism (version 9.3.1).

SARS-CoV-2 pseudovirus generation.

Single-cycle infection pseudoviruses were generated as previously described (*42*). Briefly, HEK293T cells seeded overnight in 6-well tissue culture plates (Corning) were co-transfected with the following plasmids: 1) 0.5 µg of pCDNA3.3 encoding SARS-CoV-2 spike genes with 19-residue C-terminal truncations, 2) 2 µg of MLV-based luciferase reporter gene plasmid (Vector Builder), and 3) 2 µg of MLV gag/pol (Vector Builder). The SARS-CoV-2 Omicron/BA.1 contained the following mutations relative to Wuhan-1: A67V, Δ69-70, T95I, G142D, Δ143-145, Δ211, L212I, ins214EPE, G339D, S371L, S373P, S375F, K417N, N440K, G446S, S477N, T478K, E484A, Q493K, G496S, Q498R, N501Y, Y505H, T547K, D614G, H655Y, N679K, P681H, N764K, D796Y, N856K, Q954H, N969K, L981F. Plasmids were combined with Lipofectamine 2000 (ThermoFisher Scientific) and transfected following the

manufacturer's recommendations. Culture supernatants containing SARS-CoV-2 S-pseudotyped MLV particles were collected 48 h post-transfection, aliquoted, and frozen at -80 °C for neutralization assays.

Pseudovirus neutralization assay.

HeLa-hACE2 reporter cells (BPS Bioscience Cat #79958) were seeded overnight at 10,000 cells per well in 96-well tissue culture plates (Corning). Human plasma and serum samples were heat-inactivated at 56 ˚C for 30 min. Next, monoclonal antibodies or heat-inactivated sera were serially diluted in MEM/EBSS media supplemented with 10% FBS with 50 μl of MLV viral stock and incubated for 1 h at 37 ˚C with 5% carbon dioxide. Cell culture media was removed, and cells were washed two times with PBS. The virus-antibody mixture was subsequently added to HeLa-hACE2 cells and incubated for 48 h at 37 ˚C with 5% carbon dioxide. Cells were then lysed with Luciferase Cell Culture Lysis 5× reagent (Promega), and luciferase activity was measured using the Luciferase Assay System (Promega) following manufacturer's protocols. Infectivity was measured as relative luminescence units (RLUs) using a luminometer (Perkin Elmer). The percentage neutralization was calculated as $100*(1-[RLU_{sample}-RLU_{background}]/[RLU_{isotype\ control\ mAb}-RLU_{background}])$, and the 50% neutralization concentration was interpolated from four-parameter non-linear regression fitted curves in GraphPad Prism (version 9.3.1).

FACS analysis of SARS-CoV-2 S-specific B cell responses.

Antigen-specific B cells were detected using recombinant biotinylated antigens tetramerized with fluorophore-conjugated streptavidin (SA). For detection of peripheral B cells that recognize WT and/or BA.1 RBD, 4:1 molar ratios of biotinylated antigens to SA were mixed in the following combinations: WT HexaPro S with SA-AlexaFluor 633 (Invitrogen), BA.1 HexaPro S with SA-AlexaFluor 633 (Invitrogen), WT RBD (Acro Biosystems, Cat #SPD-C82E8) with SA-BV421 (BioLegend), and BA.1 RBD (Acro Biosystems, Cat #SPD-C522e) with SA-phycoerythrin (PE; Invitrogen). For determination of subdomain reactivities within the total S-specific B cell population, antigen tetramers were mixed in the following combinations: WT HexaPro S with SA-AlexaFluor 633 (Invitrogen), BA.1 HexaPro S with SA-AlexaFluor 633 (Invitrogen), WT RBD (Acro Biosystems, Cat #SPD-C82E8) with SA-BV421 (BioLegend), BA.1 RBD (Acro Biosystems, Cat #SPD-C522e) with SA-BV421, WT NTD (Acro Biosystems, Cat #S1D-52H6) with SA-PE, BA.1 NTD (Acro Biosystems, Cat # SPD-C522d) with SA-PE, and HexaPro-stabilized WT S2 (Acro Biosystems, Cat #S2N-C52H5) with SA-BV711 (BD BioSciences). Antigen tetramers were incubated for 30 min at 4 ˚C, followed by quenching of unbound SA sites using 5 μl of 2 μM Pierce biotin (ThermoFisher Scientific). PBMCs were stained with pooled tetramerized antigens (25 nM each) and anti-human antibodies anti-CD19 (PE-Cy7; Biolegend), anti-CD3 (PerCP-Cy5.5; Biolegend), anti-CD8 (PerCP-Cy5.5; Biolegend), anti-CD14 (PerCP-Cy5.5; Invitrogen), and anti-CD16 (PerCP-Cy5.5; Biolegend) diluted in a 1:1 [v/v] mixture of Brilliant Stain Buffer (BD BioSciences) and FACS buffer (2% BSA/1 mM EDTA in 1X PBS) for 15 min on ice. Following one wash, cells were resuspended in a mixture of propidium iodide and anti-human antibodies anti-IgG (BV605; BD Biosciences), anti-IgA (FITC; Abcam), anti-CD27 (BV510; BD Biosciences), and anti-CD71 (APC-Cy7; Biolegend) and incubated for 15 min on ice. After washing two times with FACS buffer, samples were analyzed using a BD FACS Aria II (BD Biosciences).

The proportion of class-switched RBD-specific B cells that reacted with WT and/or BA.1 RBD was calculated by dividing the number of BA.1/WT cross-reactive or WT-specific IgG^+ and IgA^+ ($swIg^+$) B cells by the total number of RBD^+ S^+ $swIg^+$ B cells. The proportion of S-reactive B cells that recognized each subdomain (NTD, RBD, or S2) was calculated by dividing the number of IgG^+ and IgA^+ ($swIg^+$) B cells that recognize both S and the subdomain by the total number of S^+ $swIg^+$ cells.

Single B cell sorting.

Biotinylated recombinant WT (Acro Biosystems, Cat #SPD-C82E8) and BA.1 (Acro Biosystems, Cat #SPD-C522e) RBDs were separately mixed with AlexaFluor 633-conjugated SA (SA-633; Invitrogen) and PE-SA (Invitrogen) at a 4:1 molar ratio of antigen to SA for 30 min at 4 ˚C. The four antigen-SA pairs were then pooled to create a mixture of PE- and APC-labelled WT and BA.1 RBD tetramers. PBMCs were stained with tetramerized antigens (25 nM each) and a mixture of anti-CD19 (PE-Cy7; Biolegend), anti-CD20 (BV711; Biolegend) anti-CD3 (PerCP-Cy5.5; Biolegend), anti-CD8 (PerCP-Cy5.5; Biolegend), anti-CD14 (PerCP-Cy5.5; Invitrogen), and anti-CD16 (PerCP-Cy5.5; Biolegend) antibodies diluted in FACS buffer (2% BSA/1 mM EDTA in 1X PBS) for 15 min on ice. Stained cells were centrifuged for 10 min at 400 x *g,* washed once with FACS buffer, and centrifuged again to pellet the cells. Next, cells were resuspended in propidium iodide and anti-human antibodies anti-IgM (BV421; BD Biosciences), anti-IgG (BV605; BD Biosciences), anti-IgA (FITC; Abcam), anti-CD27 (BV510; BD Biosciences), and anti-CD71 (APC-Cy7; Biolegend) diluted in Brilliant Stain Buffer (BD BioSciences) and FACS buffer. Following 15 min of incubation on ice, Cells were washed two times, resuspended in FACS buffer, and analyzed using a BD FACS Aria II (BD Biosciences). Class-switched B cells, defined as $CD19^+CD3^-CD8^-CD14^-CD16^-PI^-IgM^-$ and IgG^+ or IgA^+, that specifically bound to the WT/BA.1 RBD mixture were single-cell index sorted into 96-well polystyrene microplates (Corning) containing 20 µl lysis buffer per well [5 µl of 5X first strand SSIV cDNA buffer (Invitrogen), 1.25 µl dithiothreitol (Invitrogen), 0.625 µl of NP-40 (Thermo Scientific), 0.25 µl RNaseOUT (Invitrogen), and 12.8 µl dH2O]. Plates were then frozen at -80 ˚C before further downstream processing.

Binding affinity measurements by biolayer interferometry.

All steps were performed at 25 ºC and at an orbital shaking speed of 1000 rpm. All reagents were formulated in PBSF buffer (PBS with 0.1% w/v BSA). Recombinant biotinylated antigens were diluted (100 nM) in PBSF and loaded onto streptavidin biosensors (Sartorius) to a sensor response of 0.6-1.0 nm and then allowed to equilibrate in PBSF for a minimum of 30 min. After a 60 s baseline step in PBSF, antigen-loaded sensors were exposed (180 s) to Fab or IgG fragments (100 nM) and then dipped (180 s) into PBSF to measure any dissociation of the antigen from the biosensor surface. Fab binding data with detectable binding responses (>0.1 nm) were aligned, inter-step corrected (to the association step) and fit to a 1:1 binding model using the FortéBio Data Analysis Software, version 11.1.

Epitope binning by biolayer interferometry.

Antibody competition with recombinant human ACE2 and comparator antibodies for binding to SARS-CoV-2 RBD was determined by BLI using a ForteBio Octet HTX (Sartorius). All binding steps were performed at 25 ºC and at an orbital shaking speed of 1000 rpm. All

reagents were formulated in PBSF (1X PBS with 0.1% w/v BSA). For ACE2 competition experiments, test antibodies (100 nM) were captured onto anti-human IgG capture (AHC) biosensors (Molecular Devices) to a sensor response of 1.0 nm-1.4 nm. IgG-loaded sensors were then soaked (20 min) in an irrelevant IgG1 solution (0.5 mg/ml) to block remaining Fc binding sites, followed by a 30 min incubation in PBSF. To assess any potential cross interactions between sensor-loaded IgG and ACE2, the IgG-loaded and blocked sensors were exposed (90 s) to a 300 nM ACE2 (Sino Biological, Cat# 10108-H08H). Sensors were next allowed to baseline (60 s) before exposing (180 s) to recombinant SARS-CoV-2 RBD (100 nM; Acro Biosystems, Cat # SPD-C52H3) and then exposed (180 s) to ACE2 (300 nM). Increased sensor responses following ACE2 exposure represented a non-ACE2-competitive binding profile, whereas antibodies showing unchanged sensor responses were designated as ACE2-competitive. Antibody competition with comparator antibodies (REGN10933, ADI-62113, COV2-2130, REGN10987, and S309) was performed using the same method as described above but with a different assay orientation: comparator antibodies were captured to anti-human IgG capture biosensors (Molecular Devices) and then exposed to antibodies of interest (300 nM) in solution.

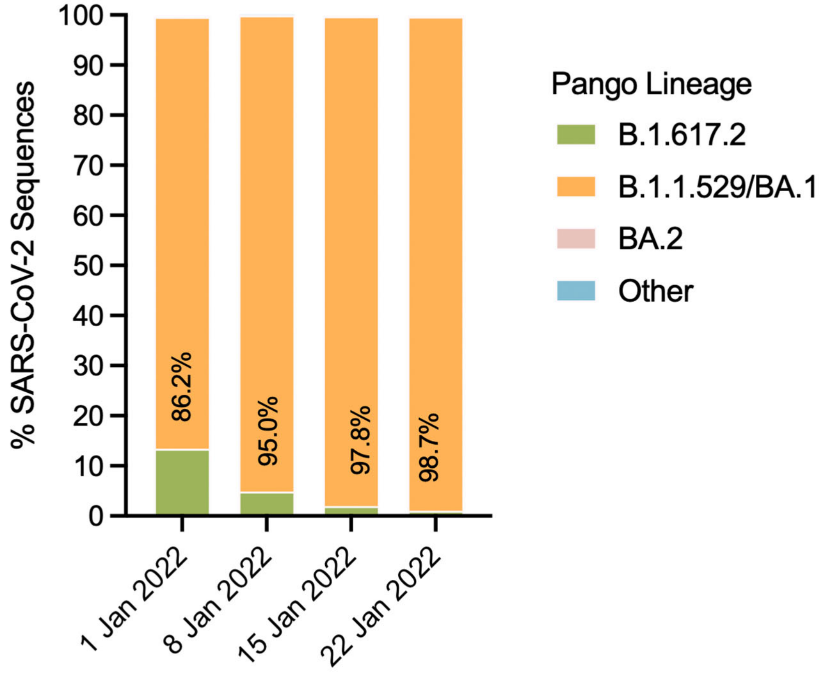

Fig. S1. Prevalence of circulating SARS-CoV-2 variants. Genomic sequencing analyses of SARS-CoV-2 infections occurring in the United States CDC Region 1 (Connecticut, Maine, Massachusetts, New Hampshire, Rhode Island, and Vermont) are colored by the indicated Pango lineage. Data was obtained using https://covid.cdc.gov/covid-data-tracker/#variant-proportions (accessed March 18, 2022).

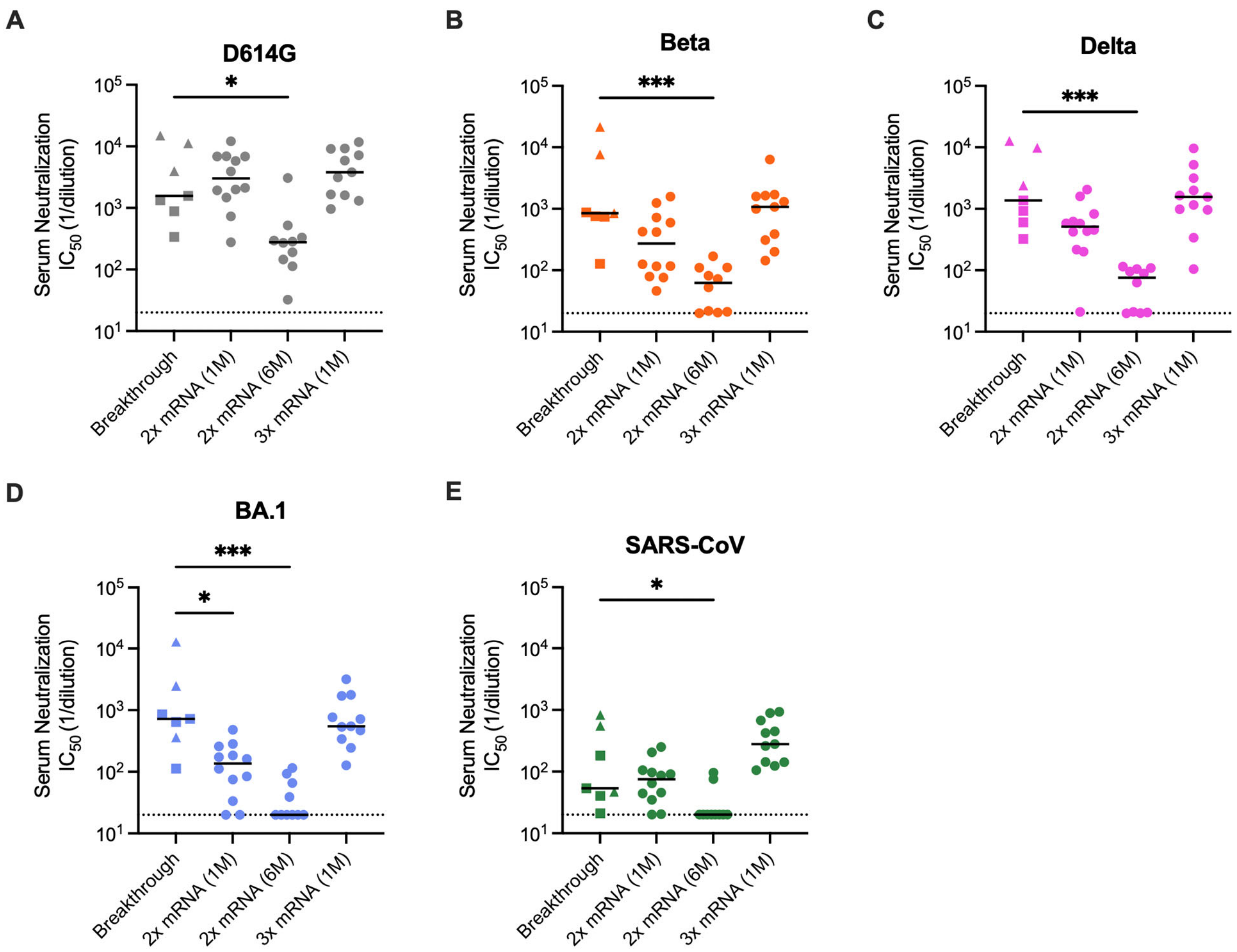

Fig. S2. Serum neutralizing activity against SARS-CoV-2 VOCs and SARS-CoV. (A-E) Serum neutralization was assessed against SARS-CoV-2 (A) D614G, (B) Beta, (C) Delta, (D) BA.1, and (E) SARS-CoV, using an MLV-based pseudovirus assay. Uninfected/vaccinated donor samples collected at one month or six months following primary vaccination (2x mRNA) or one month following mRNA booster vaccination (3x mRNA) are included for reference. Donors with breakthrough infections occurring after primary mRNA vaccination are shown as squares and those infected after booster mRNA vaccination are shown as triangles. Statistical comparisons were determined by two-sided Kruskal-Wallis test followed by Dunn's multiple comparisons. 1M, one month; 6M, six months; IC_{50}, 50% inhibitory concentration. *$P < 0.05$, **$P < 0.01$, ***$P < 0.001$.

A

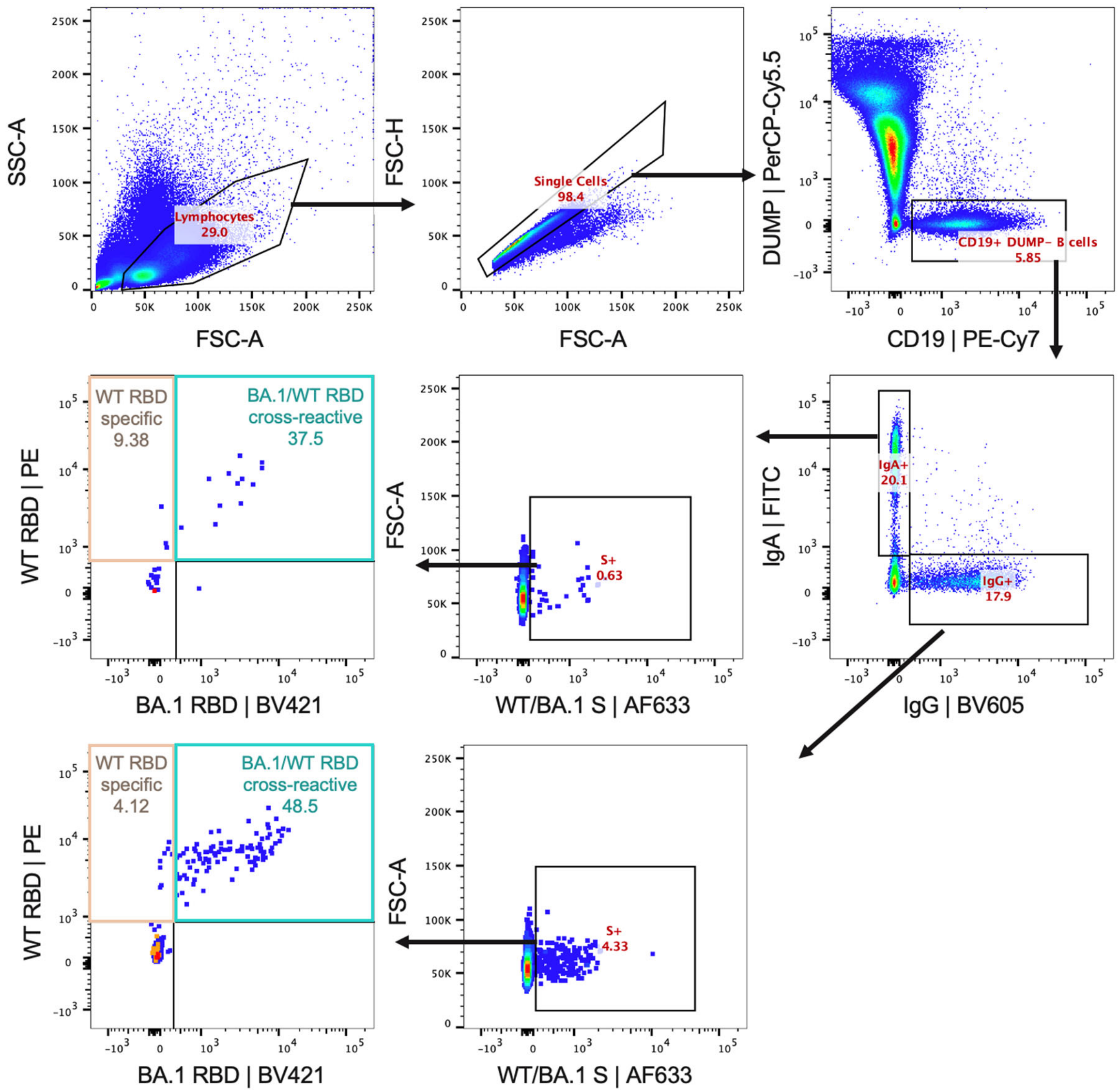
SSC-A
FSC-A
Lymphocytes
29.0
FSC-H
FSC-A
Single Cells
98.4
DUMP | PerCP-Cy5.5
CD19 | PE-Cy7
CD19+ DUMP- B cells
5.85
IgA | FITC
IgG | BV605
IgA+
20.1
IgG+
17.9
FSC-A
WT/BA.1 S | AF633
S+
0.63
WT RBD | PE
BA.1 RBD | BV421
WT RBD
specific
9.38
BA.1/WT RBD
cross-reactive
37.5
FSC-A
WT/BA.1 S | AF633
S+
4.33
WT RBD | PE
BA.1 RBD | BV421
WT RBD
specific
4.12
BA.1/WT RBD
cross-reactive
48.5

B

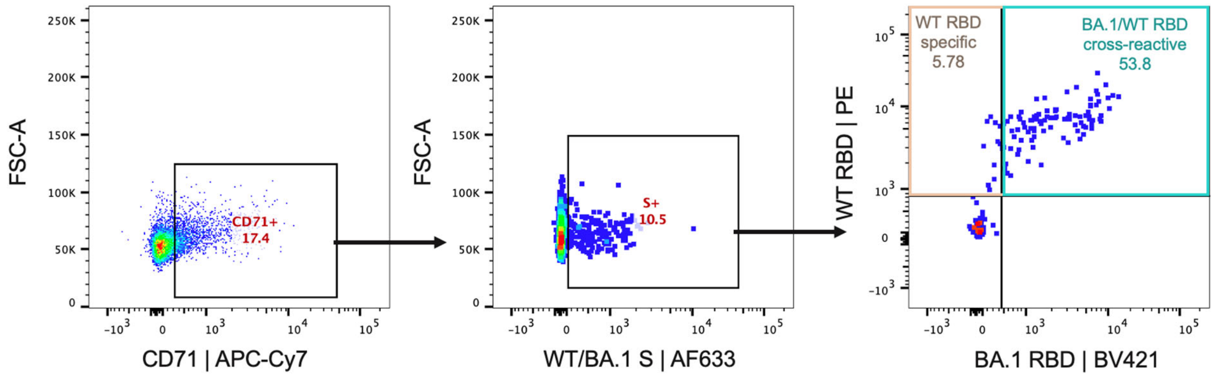
FSC-A
CD71 | APC-Cy7
CD71+
17.4
FSC-A
WT/BA.1 S | AF633
S+
10.5
WT RBD | PE
BA.1 RBD | BV421
WT RBD
specific
5.78
BA.1/WT RBD
cross-reactive
53.8

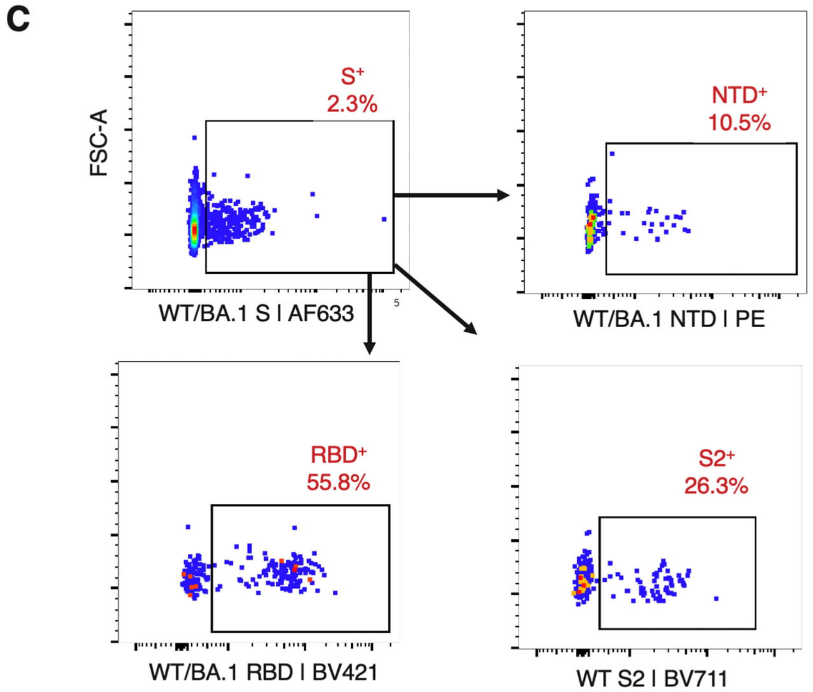

Fig. S3. FACS gating strategy for SARS-CoV-2 antigen-specific B cell staining. (A) Representative FACS gating strategy to determine frequencies of WT- and BA.1-RBD-reactive B cells among IgG^+ and IgA^+ B cells. **(B)** Representative FACS gating strategy to determine the proportion of $CD71^+S^+RBD^+$ cells that are WT-specific or BA.1/WT cross-reactive. **(C)** Representative FACS gating strategy used to calculate the proportion S-specific B cells directed to the NTD, RBD, and S2 subdomains. FSC-A, forward scatter area; FSC-H, forward scatter height; SSC-A, side scatter area.

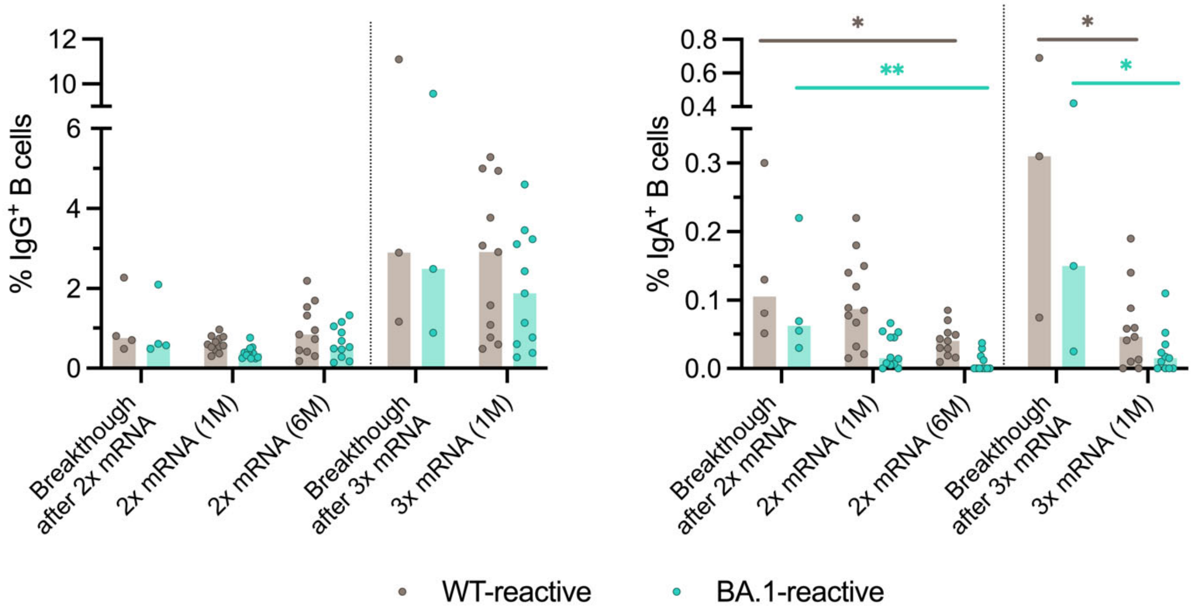

Fig. S4. Frequency of RBD-directed IgG and IgA B cells. Frequency of B cells that recognize recombinant WT and BA.1 RBDs among (left) IgG^+ and (right) IgA^+ B cells in donors experiencing BA.1 breakthrough infection after primary vaccination (2x mRNA) or booster vaccination (3x mRNA), as determined by flow cytometry. Uninfected/vaccinated donor samples collected at one month or six months following primary vaccination or one month following mRNA booster vaccination are included for reference. Bars indicate median frequencies. Statistical comparisons were determined by Kruskal-Wallis test with subsequent Dunn's multiple comparisons and two-sided Mann Whitney U test for two-dose and three-dose vaccinated individuals, respectively. 1M, one month; 6M, six months; WT, wild type. *$P < 0.05$, **$P < 0.01$.

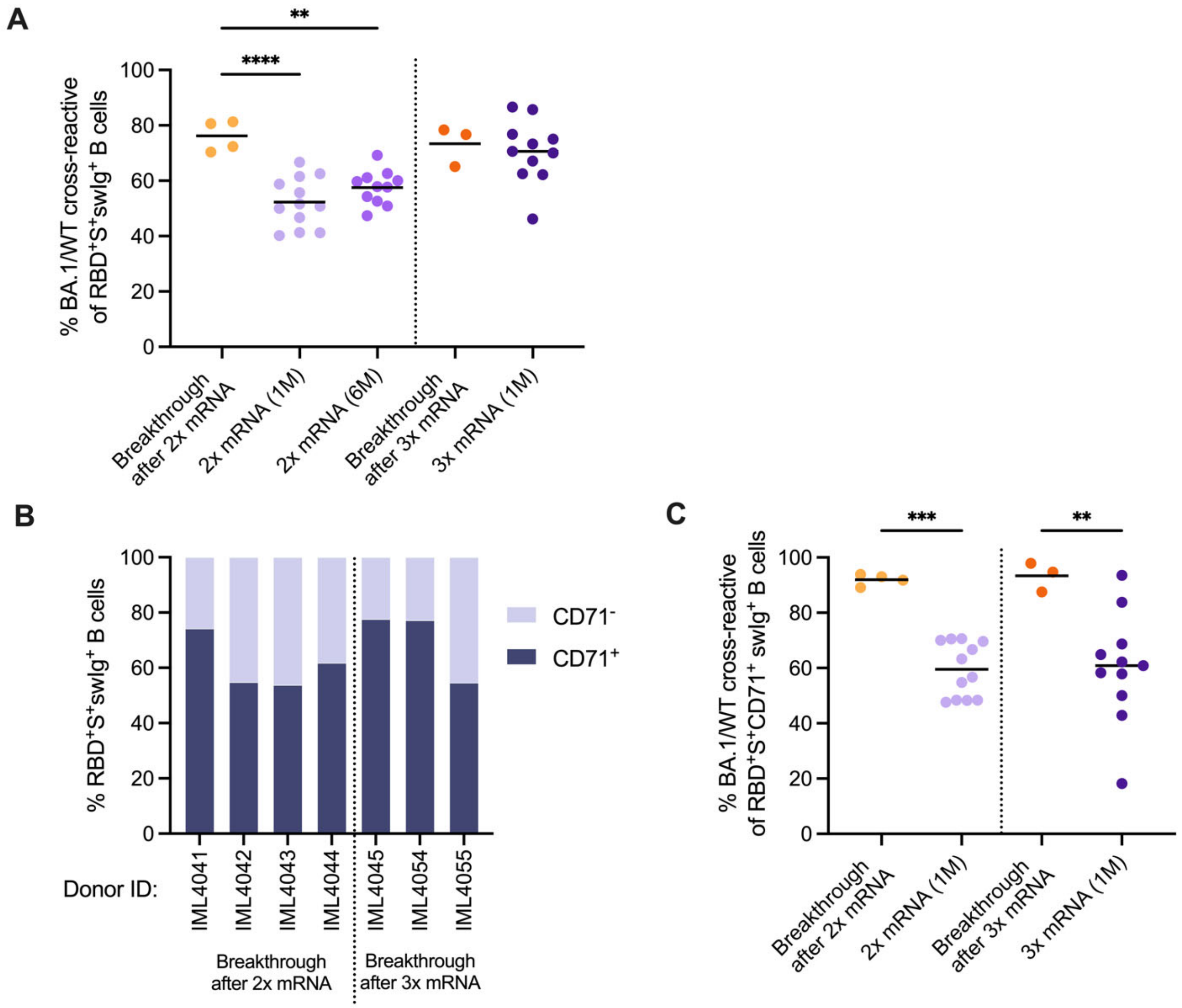

Fig. S5. Cross-reactivity of the BA.1 activated B cell response. (A) Percentage of anti-RBD B cells that cross-react with WT and BA.1 RBD among total S$^+$swIg$^+$ B cells, as determined by flow cytometry. Black bars indicate medians. **(B)** CD71 expression among RBD-directed B cells following BA.1 breakthrough infection. **(C)** Percentage of WT/BA.1 RBD cross-reactive B cells among activated CD71$^+$ B cells (S$^+$ swIg$^+$ CD71$^+$B cells). Samples collected six months after mRNA vaccination were excluded from this analysis due to low numbers of RBD-specific CD71$^+$ cells at this time point. Black bars indicate medians. Statistical comparisons were determined by two-sided Kruskal-Wallis test with Dunn's multiple comparisons. 1M, one month; 6M, six months. **P < 0.01. ***P < 0.001, ****P < 0.0001.

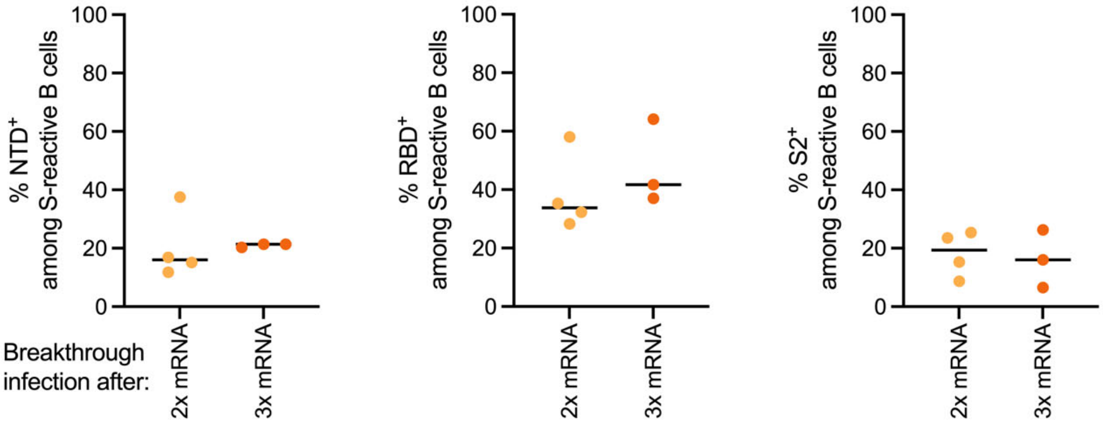

Fig. S6. B cell subdomain specificities following BA.1 breakthrough infection. Proportions of S-reactive CD71^{+} swIg^{+} B cells that recognize the NTD (left), RBD (middle), or prefusion-stabilized S2 (right) subdomains in donors who experienced breakthrough infection after either two-dose (2x) or three-dose (3x) mRNA vaccination, as determined by flow cytometry. Black bars indicate medians.

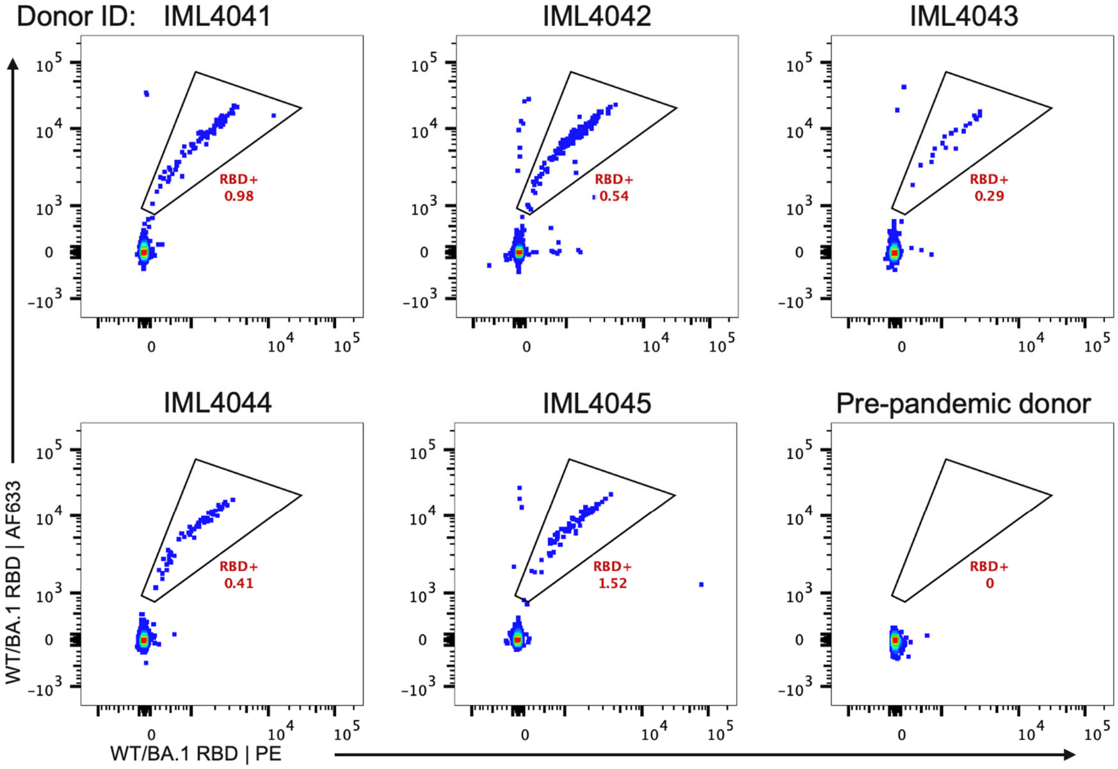

Fig. S7. FACS gating strategy for RBD-specific B cell sorting. FACS plots shown are gated on swIg$^+$ CD19$^+$ B cells. A pre-pandemic donor was included as a negative control.

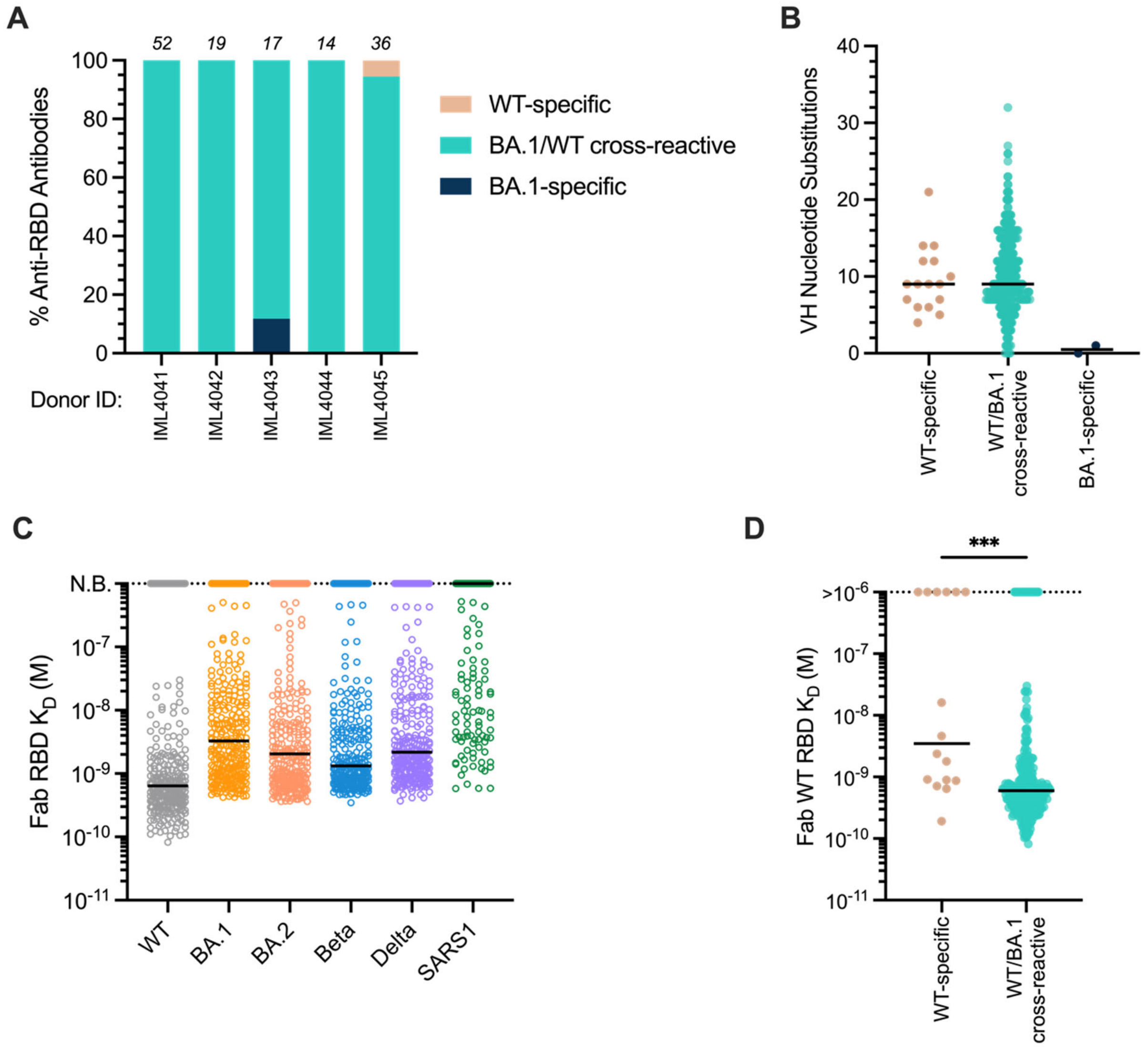

Fig. S8. Sequence and binding properties of BA.1 breakthrough infection-derived antibodies. **(A)** Binding reactivities of anti-RBD antibodies isolated from swIg^{+}CD71^{+} B cells. The number at the top of each bar represents the total number of antibodies from each donor. **(B)** VH nucleotide substitutions in WT-specific, WT/BA.1 cross-reactive, and BA.1-specific antibodies. Black bars indicate median values. **(C)** Fab binding affinity of BA.1-reactive antibodies to SARS-CoV-2 WT and VOCs and SARS-CoV RBD, as determined by BLI. Black lines represent median affinities. Antibodies with no detectable Fab binding, including those that only bind avidly, are plotted as non-binding (N.B.) on the dotted line. **(D)** Fab binding affinity to WT RBD, as determined by BLI. Black bars indicate median values. Statistical comparisons were made by two-sided Mann Whitney U test. K_D, equilibrium dissociation constant; N.B., non-binding; VH, variable heavy chain. ***P<0.001.

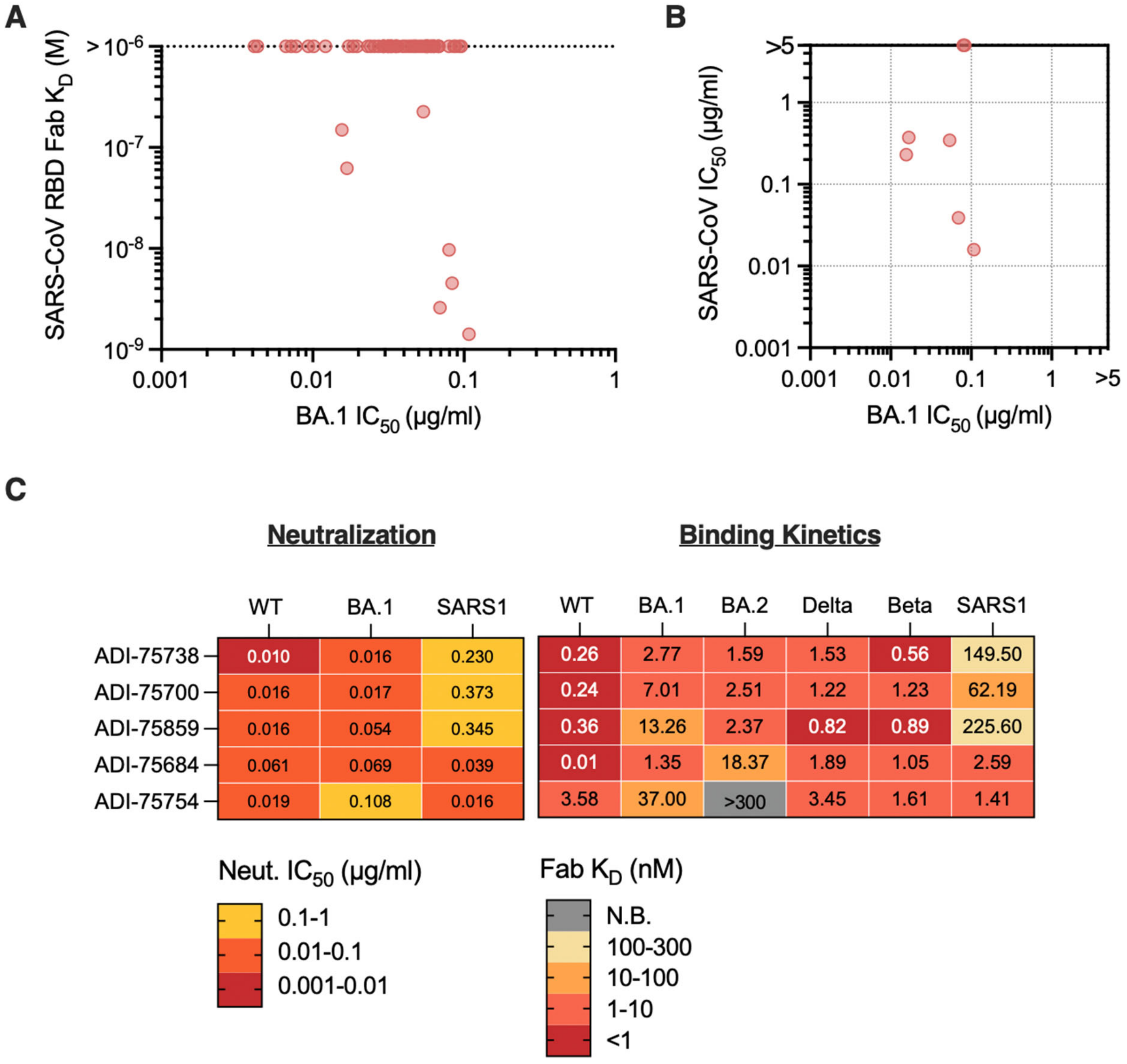

Fig. S9. Breadth of activity of SARS-CoV cross-reactive antibodies. (A) Fab binding affinities of BA.1 neutralizing antibodies to SARS-CoV RBD, as determined by BLI. **(B)** Neutralization IC_{50}s against SARS-CoV and BA.1 among antibodies displaying Fab binding to SARS-CoV. **(C)** Neutralization potency (left) and Fab binding affinity (right) of SARS-CoV cross-neutralizing antibodies. IC_{50}, 50% inhibitory concentration; K_D, equilibrium dissociation constant; N.B., non-binding; WT, wild-type.

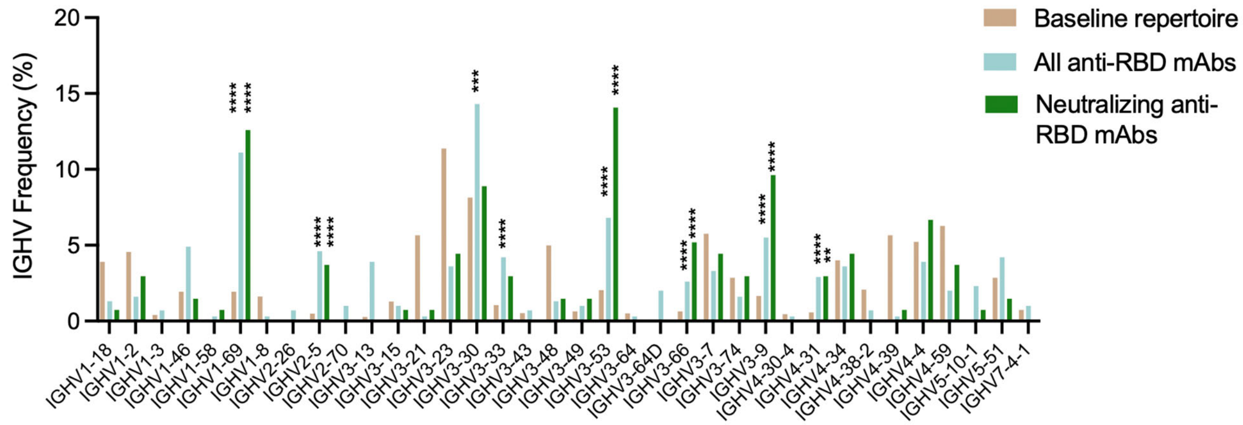

Fig. S10. IGHV germline gene usage among BA.1-neutralizing antibodies. Human IGHV germline distribution frequencies among BA.1 neutralizing antibodies, all RBD binding antibodies isolated from BA.1 breakthrough infection donors, and the human baseline (unselected) repertoire (*22*). Statistical comparisons were made by Fisher's exact test compared to the baseline repertoire. IGHV, immunoglobulin heavy variable domain. $^{**}P < 0.01$, $^{***}P < 0.001$, $^{****}P < 0.0001$.

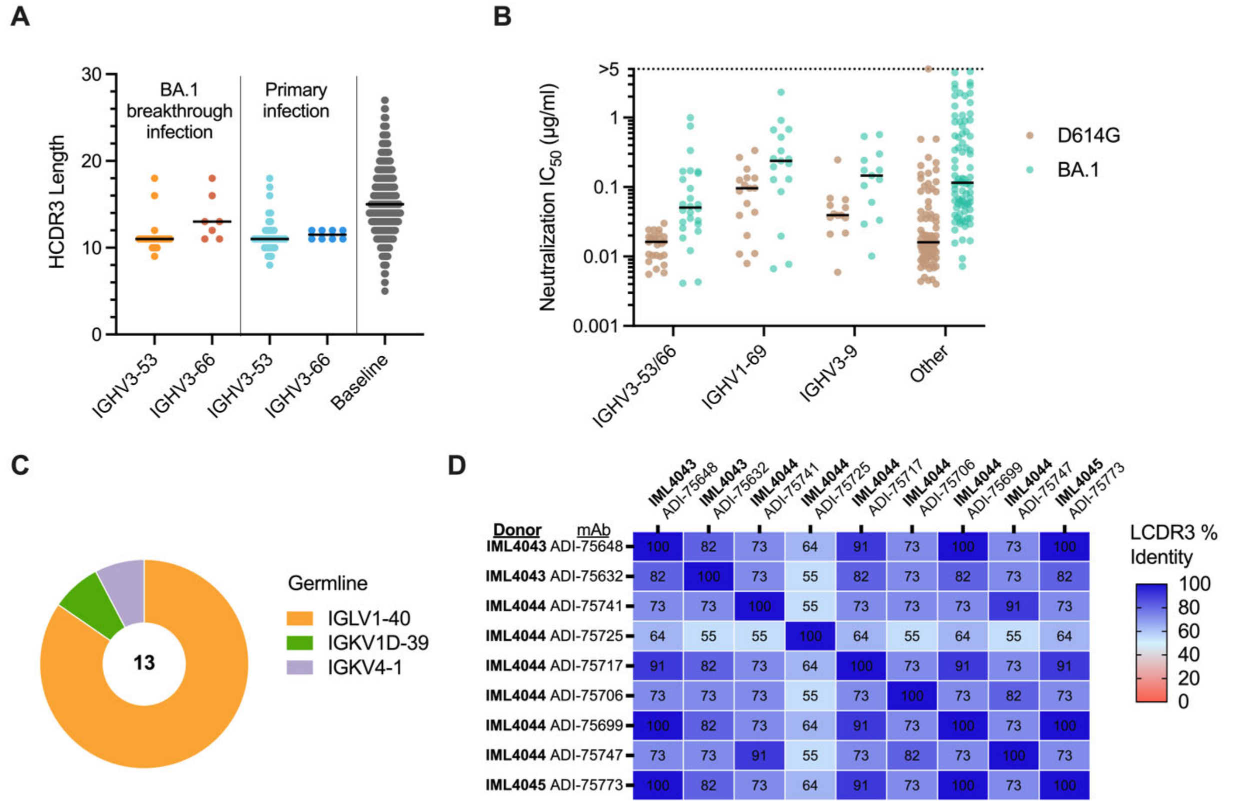

Fig. S11. Sequence and functional properties of convergent antibodies. (A) HCDR3 amino acid lengths of BA.1-neutralizing IGHV3-53/66 antibodies. HCDR3 lengths of neutralizing IGHV3-53/66 antibodies isolated following primary D614G infection and the baseline human antibody repertoire were included for comparison (*21, 22*). **(B)** Neutralizing activity of convergent IGHV germline antibodies against D614G and BA.1, as measured using an MLV-based pseudovirus assay. Black bars represent median IC_{50}s. **(C)** Light chain germline gene usage among BA.1-neutralizing, ACE2 non-competitive IGHV1-69 antibodies. The total number of antibodies is shown in the center of the pie. **(D)** Pairwise comparisons of LCDR3 amino acid identity in nine IGHV1-69/IGLV1-40-paired antibodies with the same LCDR3 lengths. The percentage identity of each pairwise comparison is shown in the middle of each square.

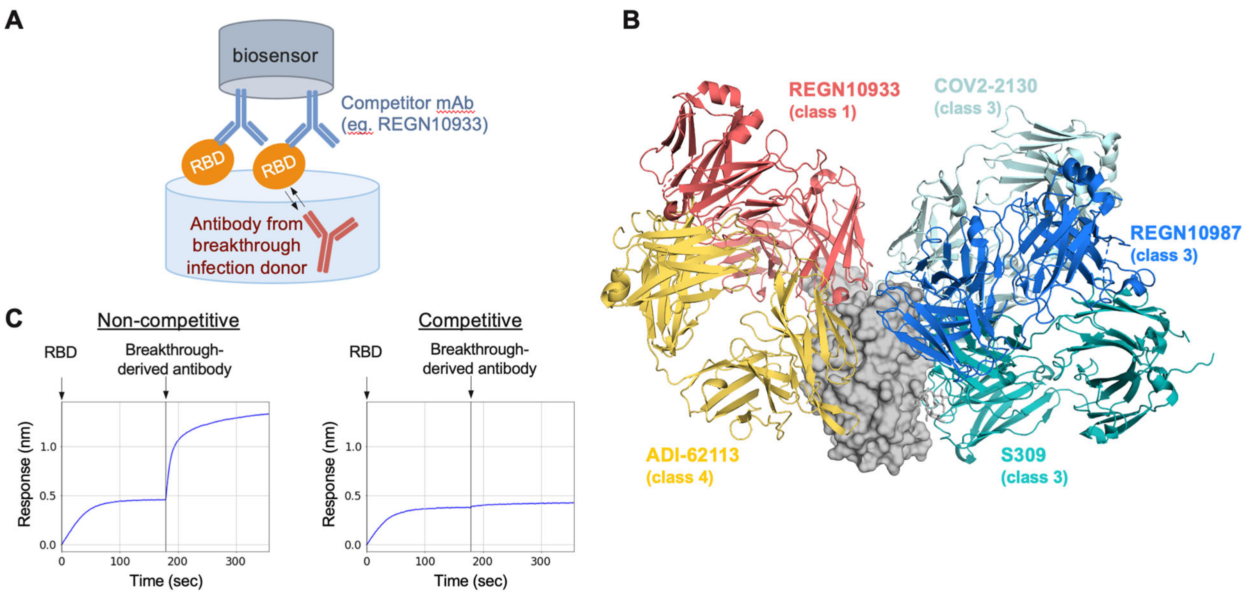

Fig. S12. Competitive binding assay schematic. (A) BLI assay set-up. Biosensors coated with competitor antibodies were loaded with recombinant WT RBD and then exposed to anti-RBD antibodies isolated from breakthrough infection donors. **(B)** Structural depiction of competitor antibodies used in BLI epitope mapping experiments in complex with the RBD (PDB: 6XDG, 7L7E, 7BEP), with each antibody class indicated in parentheses (*28, 29*). **(C)** Representative BLI plots showing competitive (left) and non-competitive (right) binding profiles.

Table S1. BA.1 breakthrough infection donor characteristics.

Donor ID	IML4041	IML4042	IML4043	IML4044	IML4045	IML4054	IML4055
Age	45	19	23	23	24	38	23
Sex	F	F	M	F	F	F	F
Vaccination History	2x BNT162b2	2x BNT162b2	2x BNT162b2	2x BNT162b2	2x BNT162b2, 1x mRNA-1273	2x mRNA-1273, 1x BNT162b2	3x BNT162b2
2nd dose vaccination date	7-May-21	22-Jul-21	23-May-21	10-Feb-21	15-May-21	5-May-21	1-May-21
3rd dose vaccination date (if applicable)	-	-	-	-	20-Dec-21	11-Dec-21	9-Dec-21
Date of PCR-confirmed infection	31-Dec-21	4-Jan-22	30-Dec-21	2-Jan-22	6-Jan-22	19-Jan-22	6-Jan-22
Days between breakthrough infection and sample collection	25	21	26	23	19	14	27

Table S2. Uninfected/mRNA-vaccinated cohort characteristics.

	2x mRNA (1M)	2x mRNA (6M)	3x mRNA (1M)
**Sample size**	12	11	11
**Age median (range)**	31 (21-42)	36 (26-42)	54 (32-58)
**Sex**			
Female (%)	9 (75%)	8 (73%)	6 (55%)
Male (%)	3 (25%)	3 (27%)	4 (36%)
Other (%)	0	0	1 (9%)
**Vaccination regimens (%)**			
	2x mRNA-1273 (75%)	2x mRNA-1273 (82%)	3x mRNA-1273 (64%)
	2x BNT162b2 (25%)	2x BNT162b2 (18%)	2x mRNA01273, 1x BNT162b2 (36%)

Table S3. Sequence, binding, and neutralization properties of isolated antibodies. (Excel datasheet)

Table S4. Raw data. (Excel datasheet)
